# Midwife or doctor leader to implement a national guideline in babies on postnatal wards (DesIGN): A cluster-randomised, controlled, trial

**DOI:** 10.1371/journal.pone.0291784

**Published:** 2023-09-28

**Authors:** Jane M. Alsweiler, Caroline A. Crowther, Jane E. Harding

**Affiliations:** 1 Department of Paediatrics: Child and Youth Health, University of Auckland, Auckland, New Zealand; 2 Liggins Institute, University of Auckland, Auckland, New Zealand; Post Graduate Institute of Medical Education and Research, INDIA

## Abstract

The aim of this trial was to determine if midwives or doctor leaders are more effective at implementing a clinical practice guideline for oral dextrose gel to treat neonatal hypoglycaemia. This was a cluster-randomised, controlled, trial. New Zealand maternity hospitals were randomised to guideline implementation by a midwife or doctor implementation leader. The primary outcome was the change in the proportion of hypoglycaemic babies (blood glucose concentration <2.6 mmol/L in the first 48 hours after birth), treated with dextrose gel from before, to three months after, implementation. Twenty-one maternity hospitals that cared for babies at risk of hypoglycaemia consented to participate, of which 15 treated babies with hypoglycaemia at both time points (7 randomised to midwifery led, 8 randomised to doctor led implementation). The primary outcome included 463 hypoglycaemic babies (292 midwifery led, 171 doctor led implementation). There was no difference in the primary outcome between hospitals randomised to midwifery or doctor led implementation (proportion treated with gel, mean(SD); midwifery led: before 71 (38)%, 3 months after 87 (12)%; doctor led: before 63 (43)%, 3 months after 86 (16)%; adjusted mean change in proportion (95%CI); 19.3% (-4.5–43.1), p = 0.11). There was an increase in the proportion of eligible babies treated with oral dextrose gel from before to 3 months after implementation of the guideline (122/153 (80%) v 144/163 (88%), OR (95%CI); 3.42 (1.67–6.98), p<0.001). Implementation of a clinical practice guideline improved uptake of oral dextrose gel. There was no evidence of a difference between midwife and doctor implementation leaders for implementing this guideline for treatment of hypoglycaemic babies. The trial was prospectively registered on the ISRCTN registry on the 20/05/2015 (ISRCTN61154098).

## Introduction

Neonatal hypoglycaemia is common, affecting half of babies with risk factors such as babies of diabetic mothers [1], and babies born small, large or preterm [2]. Neonatal hypoglycaemia may have long-term consequences including executive dysfunction [3] and impaired school performance [4, 5]. Treatment of neonatal hypoglycaemia initially involves increasing enteral feeding with formula, and, if necessary, admission to the neonatal intensive care unit for intravenous dextrose, separating mother and baby. Recently, oral dextrose gel has been shown to be effective at reversing hypoglycaemia, and also reducing neonatal intensive care (NICU) admission for hypoglycaemia and the rate of formula feeding at two weeks of age [6], and has been recommended as a first line treatment of hypoglycaemia in late preterm and term babies [7]. A New Zealand evidence-based clinical practice guideline was developed as a first step in implementing this new treatment approach [8], since a change in midwifery and neonatal practice was unlikely to occur rapidly without an active implementation strategy [9].

The most effective strategy to implement new treatments for otherwise well babies on the postnatal wards, who are usually under midwifery care, is unknown. New Zealand has a unique health care system where lead maternity carers, the majority of whom are midwives, provide most primary neonatal care [10]. Midwives are responsible for ensuring neonates in their care who are at risk are screened for neonatal hypoglycaemia and can prescribe oral dextrose gel to treat neonatal hypoglycaemia. Doctors usually only become involved in hypoglycaemic babies’ care once they receive a referral from a midwife because of a low blood glucose concentration. However, doctors would usually be approached to implement a guideline for a neonatal treatment [11].

When guidelines affect several clinical disciplines, it is not known from which discipline the leader of a guideline implementation should be drawn. Multi-disciplinary team involvement improves guideline implementation [12] and non-physician disciplines such as nurses and midwives have successfully implemented guidelines [13, 14]. However, previous implementation trials have compared implementation leaders with no intervention, or in addition to one or more interventions [15], rather than comparing one clinical discipline to another. Doctors may be perceived as being the optimal implementation leaders due to their medical knowledge and the traditional professional hierarchy that places doctors above nurses and midwives [16]. However, in a postnatal ward setting a midwife local implementation leader is likely to know more of the people who will be caring for babies at risk of hypoglycaemia and to be experienced at caring for babies at risk of hypoglycaemia.

We aimed to determine if midwives or doctors are more effective leaders for implementing a clinical practice guideline for oral dextrose gel to treat neonatal hypoglycaemia. Our hypothesis was that midwives are the most effective leaders for implementing a guideline for use of oral dextrose gel to treat neonatal hypoglycaemia in babies on postnatal wards.

## Methods

### Study design and participants

In this multi-centre, cluster, randomised controlled trial, maternity hospitals in New Zealand were randomised to having a midwife or doctor lead the implementation of the clinical practice guideline on oral dextrose gel to treat neonatal hypoglycaemia. Cluster randomisation was chosen because the intervention was implemented at the level of maternity hospitals rather than individual babies. Maternity hospitals were eligible if they provided care for babies at risk of neonatal hypoglycaemia (infant of a diabetic, late preterm, small or large for gestational age). Hospitals were not eligible if there was no doctor (paediatrician or general practitioner) available to provide medical treatment or no midwifery care for newborn babies. Primary maternity hospitals that based their neonatal guidelines on the practice of their local secondary or tertiary hospital were not eligible. Babies were included in the data collection if they developed neonatal hypoglycaemia (blood glucose concentration <2.6 mmol/L or another threshold if used by the hospital) diagnosed in the first 48 hours after birth, were not admitted to NICU at the time of the hypoglycaemia, and were eligible to receive oral dextrose gel according to the “Oral dextrose gel to treat neonatal hypoglycaemia guideline” (≥ 35 weeks gestational age and younger than 48 hours after birth) [8].

The study protocol was approved by the Health and Disability Ethics Committee (15/NTA/31). To prevent clinicians (both midwives and doctors) who might sit on an institutional review board becoming aware of the trial, the Chief Executive Officer of each participating hospital gave written, informed consent prior to randomisation on behalf of each cluster [17]. The institutional review board of each participating hospital gave approval prior to data collection. Consent by the parents of eligible babies was waived by the ethics committee. The study protocol has been previously published [18]. The trial was prospectively registered on the ISRCTN registry on the 20/05/2015 (ISRCTN61154098).

### Randomisation

The allocation sequence was generated by a statistician using computer-generated random numbers. Eligible hospitals were stratified by type of maternity hospital (primary, secondary, tertiary) and by current use of oral dextrose gel to treat hypoglycemic babies (yes, no). The central study team included a research doctor and a research midwife. The research doctor was a neonatal paediatrician and the research midwife was a senior hospital midwife. The research doctor and research midwife were informed of the allocation sequence by password protected e-mail and followed a standard predetermined strategy to identify the implementation leader at each maternity hospital. Eligible hospitals within each stratum were randomly allocated in a 1:1 ratio to either (1) the research midwife contacting the charge midwife at each hospital and asking the charge midwife to identify and recruit a key midwife based at the hospital to lead the implementation or (2) the research doctor contacting the clinical director at each hospital and asking the clinical director to identify and recruit a key doctor based at the hospital to lead the implementation. The trial steering group were blinded to the intervention allocation. All staff in participating hospitals, including the trial participants, clinicians and outcome assessors were unaware that a randomised trial was being conducted.

### Patient and public involvement

There was concern that if clinicians were aware of the trial a competition may develop between doctors and midwives to implement the guideline effectively. Therefore, with the approval of the ethics committee, clinicians were unaware that the trial was happening. To keep clinicians unaware of the trial there was no patient or public involvement in the design or conduct of the research.

### Intervention

For hospitals randomised to a midwife implementation leader, the research midwife contacted the director of midwifery in tertiary hospitals or the midwife in charge of the delivery suite in secondary or primary hospitals and introduced the plans for the implementation of the guideline. The research midwife asked the charge midwife to nominate a senior midwifery staff member to be the local implementation leader for the guideline implementation.

For hospitals randomised to a doctor implementation leader, the research doctor contacted the clinical director of newborn services in tertiary hospitals, the clinical director of paediatrics in secondary hospitals, or the doctor providing neonatal care in primary hospitals. The research doctor introduced the plans for the implementation of the guideline and asked the local doctor to nominate a senior medical staff member to be the local implementation leader for the guideline implementation.

An implementation tool kit was provided to each local implementation leader (midwife or doctor), including: copies of the “Oral Dextrose gel to treat neonatal hypoglycaemia” guideline [8] educational materials including a PowerPoint presentation (including a video showing how to administer oral dextrose gel), flow charts, posters, and pocket-cards (S1 Fig), and invitations to attend a funded education day at Auckland City Hospital and for members of the guideline development team to visit the local hospital. At the education days evidence-based information was given by a neonatal paediatrician (JA) on neonatal hypoglycaemia, oral dextrose gel to treat neonatal hypoglycaemia, and the recommendations of the “Oral dextrose gel to treat neonatal hypoglycaemia” guideline. The tool kits were distributed to each local implementation leader (midwife or doctor) at the education day and education was given on the use of the components of the tool kit.

### Study outcomes

Outcomes were measured at three different time periods: 4-week period prior to implementation, 4-week period beginning 3 months after implementation, and 4-week period beginning 6 months after implementation of the clinical practice guideline. Before the beginning of the trial the initial protocol requirement to measure outcomes for the 4 week period beginning one month after implementation of the guideline was changed to the 4-week period beginning 6 months after implementation, as more representative of a prolonged changed in practice.

#### Primary outcome

The primary outcome of the trial was the change in the proportion of babies eligible to receive dextrose gel who were treated with dextrose gel from the 4-week period before implementation of the dextrose guideline to the 4-week period beginning 13 weeks (3 months) after implementation.

#### Secondary outcomes

For randomised hospitals: the change in proportion of eligible babies treated with dextrose gel from the 4-week period prior to implementation to a 4-week period beginning at 26 weeks (6 months) after implementation.

For babies recruited at participating hospitals: the proportion of eligible hypoglycaemic babies: treated with dextrose gel; admitted to NICU (overall and for hypoglycaemia); given formula as a treatment for hypoglycaemia during hospital admission; breast feeding at discharge (full or exclusive) and adherence to “Oral dextrose gel to treat neonatal hypoglycaemia guideline” (clinical recommendation alone and clinical recommendation and practice points).

For hypoglycaemic episodes, defined as one or more sequential blood glucose concentration <2.6 mmol/L: the proportion treated with oral dextrose gel (one or more uses of oral dextrose gel) and treated with formula.

After implementation of the guideline, the local implementation leader was asked to complete an online survey about the current practice at their maternity hospital for screening, diagnosis and treatment of neonatal hypoglycaemia, which strategies they used to implement the guideline, the perceived usefulness of the educational resources in the tool kit during implementation of the guideline and to identify any barriers to implementation of the guideline.

#### Statistical analysis

Assuming an intraclass coefficient (ICC) of 0.05, 20 maternity hospitals, with 20 babies recruited at each hospital, would allow us to detect an increase in the proportion of eligible babies who are treated with dextrose gel from 40% to 60%, with 80% power and an alpha level of 0.05.

We followed a prespecified statistical analysis plan with an intention-to treat approach. A pre-specified as-treated analysis was also conducted, analysing the data using the professional discipline of the local implementation leader who actually carried out the implementation of the guideline. We did not impute missing data.

The primary analysis for the primary outcome was a linear mixed regression model at the hospital level adjusted for the type of maternity hospital (primary, secondary, tertiary) and current use of oral dextrose gel to treat hypoglycaemic babies (yes, no) as stratification factors for randomisation. Repeated measures at the hospital level were taken into account using a random cluster effect. The effect of intervention was evaluated at each visit using an interaction term between randomised groups and assessment periods. The default structure in the model was used, treating hospitals as a random effect with a distinct variance component assigned to each random effect. A secondary analysis to incorporate all the data collected at a hospital and baby level was performed using a generalised linear mixed models with a logit link for binomial distribution to estimate the difference in proportion of eligible babies treated with dextrose gel between randomisation groups in each period, as well as the change between the 3 periods.

In addition to the hospital level analysis, a baby level analysis was done including all eligible babies recruited at baseline, 3 months and 6 months after implementation. The intra-cluster correlation coefficient was estimated using the between and within cluster variances estimated from the mixed model.

Adherence to the “Oral Dextrose Gel to Treat Neonatal Hypoglycaemia Clinical Practice Guideline” was defined as meeting the recommendation if every episode of hypoglycaemia was treated with oral dextrose gel while the baby was ≥ 35 weeks and <48 hours of age. Adherence to the recommendation and practice points was defined as meeting the recommendation criteria and all doses of oral dextrose gel dose were 0.5 ml/kg (+/- 20%) and a maximum of 2 doses of oral dextrose gel were given per hypoglycaemia episode and if the blood glucose concentration was ever: <1.2 mmol/L; or < 2.6 mmol/L after 2 doses of gel in one episode of hypoglycaemia; or <2.6 mM after 6 doses of gel in 48 hours, then the baby was assessed by a doctor (medical review).

As secondary analyses, potential confounding variables (reason for risk of hypoglycaemia, prioritised as infant of diabetic, late preterm, small or large for gestational age, other, none), sex, gestational age, and mode of birth (vaginal vs caesarean section)) that are closely associated with the outcomes were considered in the model if there was an evidence of group difference by chance (≥ 10%). A sensitivity analysis was conducted excluding multiples from the analysis i.e. twins were removed from the dataset and the analysis was repeated.

Statistical analyses were performed using SAS version 9.4 (SAS Institute Inc. Cary NC) The data are presented as adjusted mean difference or odds ratios (OR) with 95% confidence intervals and two-sided p-values. A two-sided p value of <0.05 was taken to be statistically significant.

## Results

**Fig 1:**
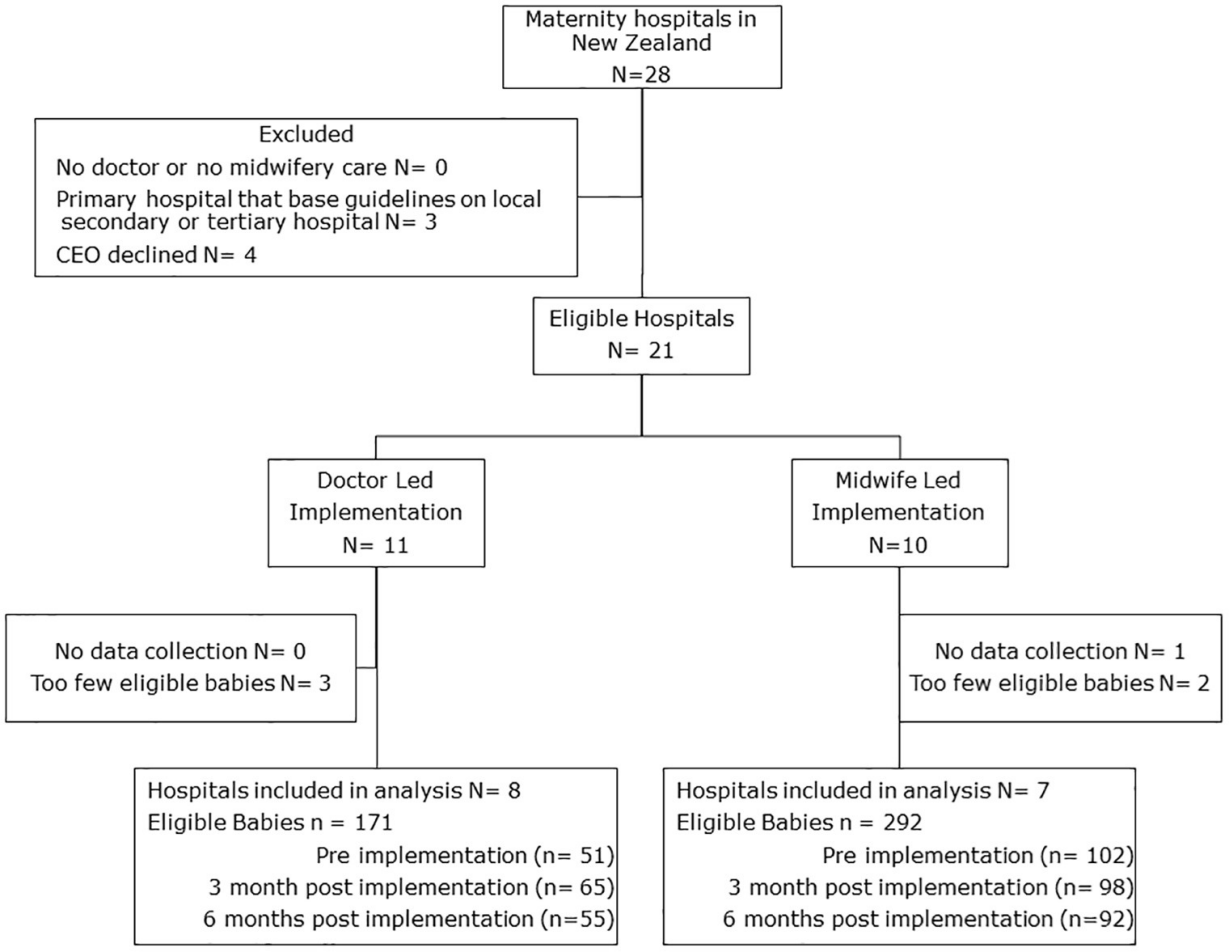
CONSORT flow chart. CEO; Chief Executive Officer.

Of the twenty-eight maternity hospitals in New Zealand, three were primary hospitals that utilised guidelines from local secondary hospitals, and four hospitals declined to participate. The Chief Executive Officer of twenty-one eligible maternity hospitals in New Zealand consented to participate and these hospitals were randomised. Of these, 15 hospitals had eligible babies at both data points for the primary outcome; 7 randomised to midwifery led and 8 randomised to doctor led implementation (Fig 1) and were included in the intention-to-treat analysis. Implementation was begun between 16 September 2015 and 11 April 2017, and data collection was between 18 August 2015 and 5 December 2017.

There were 463 eligible hypoglycaemic babies included in the analysis, 292 born in the midwifery led implementation hospitals and 171 born in the doctor led implementation hospitals. In the four weeks prior to implementation 153 babies developed hypoglycaemia, 163 babies three months after implementation and 147 babies six months after implementation of the guideline. The hospital, maternal and baby characteristics were similar between the two groups (Table 1). The mean (standard deviation (SD)) gestational age of babies who developed neonatal hypoglycaemia was 38 (2) weeks, and the birthweight z score was 0.18 (1.4). The most common risk factors for hypoglycaemia were being born to a diabetic mother or being small for gestational age.

**Table 1:** Hospital, maternal and baby characteristics of all participants.

	**Midwife Led**	**Doctor Led**				
**Hospitals**	**N = 7**	**N = 8**				
Level of care						
Tertiary	2 (29)	2 (25)				
Secondary	5 (71)	5 (63)				
Primary	0 (0)	1 (13)				
Used dextrose gel prior to trial	4 (57)	6 (75)				
Blood glucose analysis by glucose oxidase method	2 (29)	2 (25)				
	Pre-implementation		3 months post implementation		6 months post implementation	
	Midwife led	Doctor led	Midwife led	Doctor led	Midwife led	Doctor led
**Mothers**	**N = 100**	**N = 50**	**N = 97**	**N = 64**	**N = 88**	**N = 53**
Ethnicity						
Māori	17 (17)	11 (22)	15 (16)	13 (20)	11 (13)	15 (28)
Pacific Islander	5 (5)	1 (2)	6(6)	5 (8)	8 (9)	1 (2)
Asian	21 (21)	7 (14)	15 (16)	9 (14)	17 (19)	5 (9)
Other	15 (15)	7 (14)	20 (21)	9 (14)	16 (18)	7 (13)
NZ European	42 (42)	24 (48)	41 (42)	28 (44)	36 (41)	25 (47)
Diabetes in Pregnancy	33 (33)	18 (36	45 (47)	19 (30)	33 (37)	12 (23)
Caesarean Section	50 (50)	19 (38)	37 (38)	23 (38)	47 (52)	19 (36)
Multiple Pregnancy	7 (7)	2 (4)	2 (2)	3 (5)	8 (9)	5 (9)
**Babies**	**N = 102**	**N = 51**	**N = 98**	**N = 65**	**N = 92**	**N = 55**
Gestational age	37.7 (1.5)	38.2 (1.3)	37.7 (1.5(	38.5 (1.5)	37.8 (1.4)	38.3 (1.9)
Sex (male)	61 (60)	27 (53)	54 (55)	31 (48)	45 (49)	31 (56)
Birthweight (g)	3118 (680)	3214 (663)	3194 (674)	3205 (721)	3147 (727)	3200 (795)
Z score	0.17 (1.37)	0.16 (1.36)	0.36 (1.37)	0.02 (1.45)	0.17 (1.44)	0.08 (1.42)
Length (cm)	49.8 (3.0)	49.8 (3.6)	49.9 (2.81)	49.8 (3.0)	49.4 (2.8)	52.7 (3.8)
Z score	0.54 (1.27)	0.41 (1.78)	0.59 (1.23)	0.37 (1.38)	0.34 (1.15)	1.44 (1.74)
Head circumference (cm)	34.3 (1.6)	34.3 (1.6)	34.5 (1.7)	34.2 (2.0)	34.1 (1.6)	34.2 (1.9)
Z score	0.65 (1.26)	0.57 (1.30	0.83 (1.21)	0.29 (1.49)	0.46 (1.24)	0.39 (1.32)
Risk factors for hypoglycaemia						
Maternal diabetes	34 (33)	18 (35)	45 (46)	19 (29)	33 (36)	12 (22)
Late preterm	23 (23)	5 (10)	20 (21)	6 (9)	14 (15)	11 (20)
Small	26 (26)	14 (28)	16 (17)	21 (32)	30 (33)	13 (24)
Large	13 (13)	7 (14)	8 (8)	9 (14)	11(12)	7 (13)
Other	4 (4)	2 (4)	5 (5)	6 (9)	0 (0)	11 (20)
None	2 (2)	5 (10)	3 (3)	4 (6)	4 (4)	1 (2)

*Data are reported as mean (SD) or n (%). Ethnicity was prioritised: Māori, Pacific Islander, Asian, Other, NZ European. Risk factor for hypoglycaemia was prioritised: Maternal diabetes, late preterm, small, large, other, none.

Data missing–ethnicity: midwife 1; length: midwife 50, doctor 76; head circumference: midwife 45, doctor 10.

Prior to implementation of the guideline at least one hypoglycaemic baby at 12/15 (80%) of the participating hospitals received oral dextrose gel. At three- and six-months post implementation all hospitals treated at least one hypoglycaemic baby with oral dextrose gel.

There was no significant difference in the change in proportion of oral dextrose gel use from before implementation of the guideline to 3 months after implementation of the guideline (primary outcome) between hospitals randomised to midwifery led implementation and those randomised to doctor led implementation (Tables 2 and 3). There was also no difference between the groups in the change in proportion of oral dextrose use from before implementation of the guideline to 6 months after implementation. None of the hospital level secondary outcomes differed between the groups at 3 months or 6 months after implementation, including changes in proportion of admissions to NICU, the use of formula to treat hypoglycaemia, breastfeeding at discharge and adherence to the guideline (Table 3).

**Table 2:** Proportion of eligible babies treated with dextrose gel and secondary outcomes.

	Pre-implementation		3 months post implementation		6 months post implementation	
	Midwife led N = 102	Doctor led N = 51	Midwife led N = 98	Doctor led N = 65	Midwife led N = 92	Doctor led N = 55
Treated with oral dextrose gel	70.7 (37.5), 91 (38–94)	63.3 (43.0), 79 (25–100)	87 (12.1), 89 (78–100)	86.4 (16.3), (94 (72–100)	89 (12.3), 93 (76–100)	93.9 (10.3), 100 (88–100)
Admitted to NICU (any reason)	24.4 (11.1), 25 (19–33)	19.6 (22.1), 10 (0–44)	27.3 (33.5), 14 (6–33)	37.6 (34.5), 24 (13–64)	18.4 (20.3), 10 (3–40)	33.3 (21.8), 42 (17–50)
Admitted to NICU for hypoglycaemia	19.6 (8.9), 20 (13–25)	15.4 (18.1), 10 (0–27)	22.4 (36.6), 2 (0–33)	32.1 (35.4), 20 (5–54)	12.1 (16.1), 6 (0–29)	21.9 (21.3), 21 (0–42)
Formula to treat hypoglycaemia	52.6 (31.4), 50 (22–88)	27.0 (20.2), 32 (10–37)	60.3 (18.6), 67 (56–71)	38.1 (32.3), 39 (12–52)	54.6 (17.0), 50 (40–71)	45.5 (23.8), 50 (36–55)
Exclusive or fully Breastfeeding at discharge	50.1 (21.5), 50 (30–67)	47.0 (28.6), 50 (32–57)	48.3 (16.8), 56 (33–65)	47.6 (27.9), 48 (37–56)	38.0 (14.4), 40 (20–50)	57.0 (22.6), 63 (37–83)
Adherence to the guideline						
• Recommendation only	63.4 (34.5), 75 (38–88)	57.9 (43.4), 65 (17–100)	76.9 (22.2), 84 (65–89)	73.6 (24.1), 71 (54–100)	81.1 (21.0), 88 (57–100)	88.6 (17.6), 98 (82–100)
• Recommendation and Practice Points	57.1 (32.6), 70 (25–81)	52.5 (39.7), 60 (17–84)	68.4 (32.5), 78 (65–89)	65.8 (22.6), 60 (48–85)	73.3 (24.3), 80 (43–100)	77.3 (19.5), 78 (63–94)

Proportion of eligible babies treated with dextrose gel and secondary outcomes before and after implementation of the guideline in hospitals randomised to midwife and doctor led implementation (hospital level analysis)

Data are proportions of hypoglycaemic babies at each hospital, presented as mean (SD), median (IQR). NICU: neonatal intensive care unit; SD: standard deviation; IQR; Interquartile range.

**Table 3:** Primary and secondary outcomes after guideline implementation (hospital level analysis).

	Midwife led Mean (SD)	Doctor led Mean (SD)	*Adjusted mean difference	95% CI	P-value
Treated with oral dextrose gel					
3 months	16.3 (39.1)	23.0 (46.0)	19.3	-4.5–43.1	0.11
6 months	18.3 (27.8)	30.5 (39.3)	23.4	-4.0–50.8	0.09
Admitted to NICU (any reason)					
3 months	2.9 (28.9)	18.0 (32.0)	13.5	-16.4–43.4	0.35
6 months	-6.0 (13.0)	13.6 (25.5)	18.0	-11.9–47.9	0.22
Admitted to NICU for hypoglycaemia					
3 months	2.9 (35.4)	16.8 (37.5)	11.0	-21.8–43.7	0.48
6 months	-7.4 (12.8)	6.5 (22.6)	11.0	-21.7–43.7	0.48
Formula to treat hypoglycaemia					
3 months	7.7 (28.3)	11.1 (32.1)	1.1	-27.7–29.8	0.94
6 months	2.0 (19.2)	18.5 (19.7)	14.2	-14.5–42.9	0.31
Exclusive or fully breastfeeding at discharge					
3 months	-1.9 (24.1)	0.6 (30.0)	2.7	-26.1–31.5	0.84
6 months	-12.1 (17.1)	10.0 (25.2)	22.4	-6.4–51.2	0.12
Adherence to the guideline (Recommendation only)					
3 months	13.4 (43.1)	15.8 (47.4)	12.9	-19.3–45.1	0.40
6 months	17.7 (15.3)	30.8 (44.8)	23.6	-8.6–55.8	0.14
Adherence to the guideline (Recommendation and Practice Points)					
3 months	11.3 (51.4)	13.3 (48.7)	11.5	-27.8–50.9	0.54
6 months	16.1 (12.0)	24.8 (45.7)	18.2	-21.2–57.5	0.34

Primary and secondary outcomes after guideline implementation in hospitals randomised to midwife led and doctor led implementation (hospital level analysis).

Data are the change in proportion of eligible babies (%) from prior to implementation to 3 months after implementation (3 months) and to 6 months after implementation (6 months). * Adjusted for use of oral dextrose gel before implementation and accounting for repeated measures at the hospital level. The pre-specified variable, type of maternity hospital, was not included as with only one primary care hospital the model did not converge.

CI: Confidence Interval, NICU: neonatal intensive care unit; SD: standard deviation

In the baby level analysis, the estimated ICC was 0.024. There was no difference between groups in the primary or secondary outcomes at 3 months post implementation (S1 Table). At 6 months post implementation babies born at hospitals where the guideline had been implemented by doctors were more likely to be admitted to NICU than babies born at hospitals where the guideline was implemented by midwives, but not more likely to be admitted to NICU for hypoglycaemia. There were no other differences between randomisation groups. Adherence to the guideline recommendations and practice points increased from before implementation of the guideline to 3 months and 6 months after implementation (S1 Table). Overall, there was an increase in the proportion of eligible babies treated with oral dextrose gel from prior to implementation to 3 months after implementation (122/153 (80%) v 144/163 (88%), OR (95%CI); 3.42 (1.67–6.98), p<0.001).

Eleven of the implementation leaders responded to the survey (7 doctors and 4 midwives). On a 5-point Linkert scale ranging from Not Useful (1) to Extremely Useful (5) the clinical practice guideline had a median (interquartile range (IQR)) score of 5 (4–5), with no difference between doctors and midwives (doctors 5 (3–5) v midwives 4.5 (4–5), p = 0.84). From the tool kit, doctors were more likely than midwives to have used the PowerPoint presentation (4/7 (57%) v 0/3 (0%), p = 0.04) and pocket flowcharts (5/7 (71%) v 0/3 (0%), p = 0.02) to implement the guideline. Both groups reported seeking multi-disciplinary consultation and running education sessions at their local hospital as strategies to implement the guideline. Doctors were more likely to identify barriers to implementation, including a perceived lack of ability for midwives and nurses to prescribe dextrose gel and the size and cost of the dextrose gel bottles.

Of the 7 hospitals randomised to midwife led implementation, 2 actually had the implementation led by a doctor, and of the 8 randomised to doctor led, 1 had the implementation led by a midwife. However, as-treated analysis did not show any difference between the doctor and midwife led implementation (adjusted mean change in proportion (95%CI); 10.4% (-15.9–36.8), p = 0.44). There was no difference in the primary outcome when analysed by generalised lined mixed models, and neither excluding multiples nor adding mode of delivery (>10% difference) to the model affected the primary outcome.

## Discussion

In this multi-centre, cluster, randomised controlled trial to compare doctor or midwife led implementation of the “Oral dextrose gel to treat neonatal hypoglycaemia clinical practice guideline”, we found no significant difference in the primary outcome of the proportion of babies eligible to receive dextrose gel who were treated with dextrose gel from before implementation of the dextrose guideline to three months after implementation. Implementation of the guideline was associated with an increased use of oral dextrose gel to treat neonatal hypoglycaemia, and improved adherence to the guideline at both 3 months and 6 months after implementation.

There was no evidence of a difference in the effectiveness of midwives and doctor implementation leaders at implementing this guideline for treatment of babies on the postnatal ward. To our knowledge this is the first trial to investigate the efficacy of midwives for implementing evidence-based guidelines, with previous trials mainly having the implementation led by doctors [15, 19]. Midwifery-led continuity of care reduces the incidence of preterm birth, fetal loss and neonatal death [20], although an increase in adverse events has been reported [21]. In New Zealand, midwives practice independently, have prescribing rights and have experience in implementing strategies to improve breastfeeding [14]. Midwives have expertise in providing care for well babies on the postnatal wards, which makes them eligible to be considered as local implementation leaders and included in the implementation of clinical practice guidelines in this context.

This trial compared the effectiveness of implementation leaders from different clinical disciplines rather than local opinion leaders. Local opinion leaders are trustworthy and interconnected individuals likely to be persuasive agents of behavioural change, which is not a function of an individual’s position or status [22]. As the implementation leaders in our trial were appointed by management, they were champions rather than local opinion leaders. There are multiple ways to identify opinion leaders including self-selection of volunteers and expert identification [23]. However, it can be difficult for specialties other than doctors to be nominated as local opinion leaders. In a previous randomised trial to investigate local opinion leaders to improve breastfeeding rates, physicians, general physicians and midwives were asked to identify local opinion leaders. All of the chosen opinion leaders were obstetricians in senior management positions and none were midwives [24]. As our trial was investigating the effect of clinical discipline, the research doctor or midwife approached a lead clinician at each site from the relevant discipline and asked them to nominate a senior clinician from their discipline in their site to be the local implementation leader, to ensure that the local implementation leader was of the randomised discipline. Despite this, in 3 hospitals the local implementation leader who actually led the implementation was of a different discipline from that randomised, which may reflect local resourcing and hierarchies.

Implementation of the clinical practice guideline increased the use of oral dextrose gel, reduced variability between hospitals and improved adherence to the guideline recommendation and practice points. These findings agree with previous research findings that active guideline implementation improves uptake of an intervention [25]. Although implementation of the guideline increased use of oral dextrose gel, there was no improvement in clinical outcomes, with no difference in NICU admission, NICU admission for hypoglycaemia, formula use or exclusive or full breastfeeding after discharge after implementation of the guideline. A recent Cochrane systematic review found that oral dextrose gel in hypoglycaemic babies reduced the incidence of mother‐infant separation for treatment and increased the likelihood of full breast feeding after discharge [26]. The difference between our findings and those of the systematic review may reflect the limited sample size and thus power of this trial to detect a difference in the secondary outcomes.

Previously, various barriers to implementation of guidelines in different healthcare settings have been identified [25, 27]. As strategies that have identified barriers and enablers to implementation of a guideline are more likely to be successful [9] we undertook a clinician survey to identify barriers and enablers to implementation of the recommendations within the clinical practice guideline. The survey found that a guideline would be the most useful enabler and availability of oral dextrose gel the most important barrier with no difference in whether a doctor or midwife should lead the implementation [28].

A strength of this trial was that clinicians implementing the guideline and collecting the data were unaware of the trial. This was important, as if clinicians had been aware of the trial then a competition may have developed between doctors and midwives to implement the guideline effectively. As clinicians commonly participate in institutional review boards and were likely to discover the trial, the national ethics committee gave approval for locality approval at each site to be gained initially from the Chief Executive Officer of the hospital, rather than the local institutional review board, in order to keep clinicians unaware of the trial. Following randomisation and implementation of the guideline, approval was then sought from each local institutional review board for data collection at each hospital.

There were several limitations in this trial. First, the trial was underpowered as there were fewer hospitals with eligible babies at both timepoints of the primary outcome than assumed when calculating the sample size and there was a significant difference in cluster sizes. In addition, the number of eligible hypoglycaemic babies was different between the randomisation arms. Despite stratification by type of maternity hospital, several of the larger maternity hospitals were randomised to the midwifery group due to chance. These limitations mean that we had limited power to detect anything other than large differences between the two groups. Second, we based our sample size calculations on an increase from 40% of eligible babies being treated with oral dextrose gel to 60%. However, we found that prior to randomisation 67% of hospitals reported using dextrose gel, and in the pre-implementation data collection 80% of eligible hospitals were already using oral dextrose gel prior to implementation of the guideline. Although this trial had begun before the initial Cochrane Systematic Review was published [7], there was a rapid uptake of oral dextrose gel in New Zealand. This was unexpected as typically new interventions are slow to become part of routine practice [22], and require active implementation, audit and feedback to achieve high uptake [29]. The rapid uptake of oral dextrose gel as a treatment for neonatal hypoglycaemia may reflect that the randomised controlled trial which demonstrated the effectiveness of oral dextrose gel was done locally [6]. Moreover, clinicians believe that prescribing oral dextrose gel to treat neonatal hypoglycaemia is beneficial [28], and oral dextrose gel has been shown to reduce hospital costs for the management of neonatal hypoglycaemia [30]. While the initial proportion of babies treated with oral dextrose gel was higher than we anticipated, this is unlikely to have affected the results as implementation of the guideline increased use of oral dextrose gel to treat neonatal hypoglycaemia for babies born in hospitals randomised to midwifery and doctor led implementation. Third, while we surveyed implementation leaders on the usefulness of the guideline and which tools they found useful, a more in-depth mixed model process evaluation would have added value to understand and interpret the findings fully.

The results of our trial suggest that midwives and doctors are similarly effective clinical disciplines at implementing clinical practice guidelines. Further research is required on effective methods to implement guidelines for patients who are cared for by more than one clinical discipline.

## Conclusion

Implementation of clinical practice guidelines is an effective method of increasing evidence-based practice. Our implementation strategy successfully implemented a clinical practice guideline and increased the use of oral dextrose gel to treat neonatal hypoglycaemia. Midwives and doctor implementation leaders were equally effective at leading implementation of this guideline for the treatment of babies on the postnatal ward. Our findings suggest that non-medical clinical disciplines are effective options as local leaders for the implementation of clinical practice guidelines.

## Supporting information

S1 Fig Oral dextrose gel to treat neonatal hypoglycaemia: Clinical practice guidelines toolkit. Pocket flowchart and Flowchart from the Oral Dextrose Gel to treat Neonatal Hypoglycaemia: Clinical Practice Guideline Toolkit.
(DOCX)

S1 Table Eligible babies treated with oral dextrose gel and secondary outcomes (baby level analysis). (DOCX)
