# Supplementary material for: Midwife or doctor leader to implement a national guideline in babies on postnatal wards (DesIGN): A cluster-randomised, controlled, trial
Source: PLoS One. 2023 Sep 28;18(9):e0291784. doi: 10.1371/journal.pone.0291784 (PMC10538667; doi:10.1371/journal.pone.0291784)
Supplement: S1 Fig — Pocket flowchart and Flowchart from the Oral Dextrose Gel to treat Neonatal Hypoglycaemia: Clinical Practice Guideline Toolkit. (DOCX) [file pone.0291784.s001.docx]

**Oral Dextrose Gel to treat Neonatal Hypoglycaemia:**

**Clinical Practice Guidelines Toolkit**

1. **Pocket flow chart**

- **Use 0.5 ml per kg 40% oral dextrose gel for**
  - babies ≥ 35 weeks’ and <48 hours after birth
  - blood glucose concentrations 1.2 to <2.6 mM
- A second dose of gel may be given after 30 minutes
  - Seek medical review if blood glucose <2.6 mM after 2 doses of gel
- **If blood glucose <1.2 mM at any time**
  - seek urgent medical review
  - give a dose of gel whilst waiting for assistance
- Give a maximum of 6 doses of gel in 48 hours

**Hypoglycaemia**

**BGC < 1.2 mmol/L**

**BGC 1.2 to < 2.6 mmol/L**

**Urgent Medical Review**

**0.5 ml/kg Dextrose gel**

**Re-check blood glucose in 30 minutes**

**Urgent Medical Review**

**Blood glucose <2.6 mmol/L**

**BGC < 1.2 mmol/L**

**BGC 1.2 to < 2.6 mmol/L**

**Urgent Medical Review**

**0.5 ml/kg Dextrose gel**

**Re-check blood glucose in 30 minutes**

1. **Oral Dextrose Gel to Treat Neonatal Hypoglycaemia Flow Chart**

**For babies diagnosed with hypoglycaemia ≥ 35 weeks’ gestational age and younger than 48 hours after birth**

**BGC < 1.2 mmol/L**

**BGC 1.2 – 2.6 mmol/L**

**BGC ≥ 2.6 mmol/L**

**Medical Review**

**Give dextrose gel while waiting for review**

**Treatment 1: 0.5 ml/kg oral 40% dextrose gel and encourage to breast feed**

**Routine postnatal care**

**Recheck Blood Glucose Concentration (BGC) in 30 mins**

***Medical review if 6 doses of dextrose gel required in 48 hours**

**Oral dextrose gel to treat neonatal hypoglycaemia: Clinical Practice Guidelines 2015**

**Medical Review**

**Routine postnatal care**

**BGC < 2.6 mmol/L**

**BGC ≥ 2.6 mmol/L**

**Recheck Blood Glucose Concentration (BGC) in 30 mins**

**Routine postnatal care**

***Treatment 2: 0.5 ml/kg oral 40% dextrose gel and encourage to breast feed**

**Medical Review**

**Give dextrose gel while waiting for review**

**BGC < 1.2 mmol/L**

**BGC 1.2 – 2.6 mmol/L**

**BGC ≥ 2.6 mmol/L**
