# Supplementary material for: Midwife or doctor leader to implement a national guideline in babies on postnatal wards (DesIGN): A cluster-randomised, controlled, trial
Source: PLoS One. 2023 Sep 28;18(9):e0291784. doi: 10.1371/journal.pone.0291784 (PMC10538667; doi:10.1371/journal.pone.0291784)
Supplement: S1 Table — (DOCX) [file pone.0291784.s002.docx]

**S2 Table.** **Eligible babies treated with oral dextrose gel and secondary outcomes (baby level analysis).**

|  | Midwife led | Doctor led | Between group  difference  Doctor v Midwife* | | Between time  difference  All^#^ | |
| --- | --- | --- | --- | --- | --- | --- |
|  | N (%) | N (%) | Adj. OR/MD^  (95% CI) | P value | Adj. OR/MD^  (95% CI) | P value |
| Treated with oral dextrose gel | | | | | | |
| Pre-implementation | 82 (80) | 40 (78) | 0.84  (0.28-2.51) | 0.75 |  |  |
| 3 months | 88 (89) | 56 (86) | 0.66  (0.19-2.30) | 0.51 | 3.42  (1.67-6.98) | <0.001 |
| 6 months | 83 (90) | 52 (95) | 1.23  (0.26-5.71 | 0.79 | 4.31  (1.94-9.57) | <0.001 |
| Admitted to NICU (any reason) | | | | | | |
| Pre-implementation | 19 (19) | 13 (26) | 1.44  (0.46-4.55) | 0.53 |  |  |
| 3 months | 13 (13) | 16 (25) | 2.09  (0.65-6.68) | 0.21 | 0.95  (0.51-1.78) | 0.87 |
| 6 months | 15 (16) | 21 (38) | 3.14  (1.01-9.73) | <0.05 | 1.07  (0.60-1.94) | 0.81 |
| Admitted to NICU for hypoglycaemia | | | | | | |
| Pre-implementation | 15 (15) | 10 (20) | 1.42  (0.43-4.69) | 0.56 |  |  |
| 3 months | 9 (9) | 11 (17) | 2.15  (0.62-7.51) | 0.23 | 0.90  (0.45-1.80) | 0.77 |
| 6 months | 9 (10) | 12 (22) | 2.85  (0.82-9.89) | 0.10 | 0.77  (0.39-1.51) | 0.44 |
| Formula to treat hypoglycaemia | | | | | | |
| Pre-implementation | 57 (56) | 16 (31) | 0.29 (0.09-0.92) | 0.04 |  |  |
| 3 months | 58 (59) | 25 (39) | 0.37  (0.12-1.14) | 0.08 | 1.25  (0.78-2.01) | 0.35 |
| 6 months | 53 (58) | 24 (44) | 0.64  (0.21-1.95) | 0.43 | 1.40  (0.86-2.28) | 0.17 |
| Exclusive or fully breastfeeding at discharge | | | | | | |
| Pre-implementation | 40 (39) | 23 (45) | 1.52  (0.60-3.83) | 0.38 |  |  |
| 3 months | 42 (43) | 31 (48) | 1.45  (0.59-3.59) | 0.42 | 1.06  (0.66-1.70) | 0.80 |
| 6 months | 33 (36) | 25 (46) | 1.80  (0.72-4.52) | 0.21 | 0.82  (0.50-1.32) | 0.41 |
| Adherence to the guideline  (Recommendation only) | | | | | | |
| Pre-implementation | 72 (71) | 38 (75) | 1.28  (0.37-4.38) | 0.70 |  |  |
| 3 months | 79 (81) | 47 (72) | 0.60  (0.17-2.09) | 0.42 | 2.09  (1.16-3.74) | 0.01 |
| 6 months | 75 (82) | 50 (91) | 1.47  (0.35-6.14) | 0.60 | 2.96  (1.55-5.67) | 0.001 |
| Adherence to the guideline (Recommendation and Practice Points) | | | | | | |
| Pre-implementation | 66 (65) | 34 (67) | 1.06  (0.42-2.69) | 0.89 |  |  |
| 3 months | 74 (76) | 44 (68) | 0.63  (0.25-1.62) | 0.34 | 1.94  (1.14-3.29) | 0.01 |
| 6 months | 67 (73) | 44 (80) | 1.07  (0.40-2.87) | 0.90 | 1.93  (1.12-3.32) | 0.02 |
| Hypoglycaemic episodes mean (SD) | | | | | | |
| Pre-implementation | 1.4 (0.8) | 1.3 (0.65) | 0.98  (0.72-1.32) | 0.88 |  |  |
| 3 months | 1.4 (0.7) | 1.4 (0.6) | 1.03  (0.78-1.36) | 0.83 | 1.04  (0.86-1.26) | 0.69 |
| 6 months | 1.3 (0.6) | 1.4 (0.6) | 0.96  (0.72-1.29) | 0.81 | 1.00  (0.82-1.22) | 0.98 |
| Hypoglycaemic episodes treated with dextrose gel | | | | | | |
| Pre-implementation | 1.0 (0.7) | 1.0 (0.7) | 1.04  (0.73-1.47) | 0.83 |  |  |
| 3 months | 1.2 (0.7) | 1.1 (0.6) | 0.99  (0.73-1.36) | 0.97 | 1.21  (0.97-1.50) | 0.09 |
| 6 months | 1.2 (0.6) | 1.2 (0.7) | 0.99  (0.72-1.36) | 0.96 | 1.22  (0.98-1.52) | 0.07 |
| Hypoglycaemic episodes treated with formula | | | | | | |
| Pre-implementation | 0.8 (0.9) | 0.4 (0.8) | 0.54  (0.29-1.02) | 0.06 |  |  |
| 3 months | 0.7 (0.8) | 0.5 (0.7) | 0.64  (0.35-1.16) | 0.14 | 1.04  (0.78-1.37) | 0.81 |
| 6 months | 0.7 (0.7) | 0.6 (0.7) | 0.89  (0.49-1.60) | 0.69 | 1.02  (0.76-1.36) | 0.90 |
| Total amount of dextrose gel per baby (ml/kg) | | | | | | |
| Pre-implementation | 0.6 (0.5) | 0.6 (0.5) | 0.03  (-0.13-0.20) | 0.68 |  |  |
| 3 months | 0.7 (0.5) | 0.7 (0.4) | 0.04  (-0.12-0.20) | 0.61 | 0.09  (-0.02-0.19) | 0.11 |
| 6 months | 0.7 (0.5) | 0.8 (0.5) | 0.09  (-0.07-0.26) | 0.26 | 0.13  (0.03-0.24) | 0.09 |

Eligible babies treated with oral dextrose gel, and secondary trial outcomes, in those born in hospitals randomised to midwife led or doctor led implementation at each of three time points (baby level analysis).

Data are n (%), mean (SD), odds ratio/mean difference (95% confidence interval)

* Comparison of babies born in hospitals randomised to midwifery or doctor led implementation prior to implementation, 3 months after implementation and 6 months after implementation

# Comparison of each outcome in all babies over time: prior to implementation to 3 months after implementation (3 months) and to 6 months after implementation (6 months).

^ Adjusted for use of oral dextrose gel before implementation and hospital type

Pre implementation (midwife led n = 102, doctor led n = 51), 3 months post implementation (midwife led n = 98, doctor led n = 65), 6 months post implementation (midwife led n = 92, doctor led n = 55)
