# Supplementary material for: Midwife or doctor leader to implement a national guideline in babies on postnatal wards (DesIGN): A cluster-randomised, controlled, trial
Source: PLoS One. 2023 Sep 28;18(9):e0291784. doi: 10.1371/journal.pone.0291784 (PMC10538667; doi:10.1371/journal.pone.0291784)
Supplement: Attachment — Submitted filename: Response to reviewers.docx [file pone.0291784.s003.docx]

30^th^ June 2022

Dear Editor,

Thank you for your email of 11^th^ May 2022 and for providing us the opportunity to revise our manuscript " Midwife or doctor leader to implement a national guideline in babies on postnatal wards (DesIGN): a cluster-randomised, controlled, trial. " in response to the comments received.

We have revised the manuscript in response to the editor’s comments and have provided a response to each of the individual queries.

Many thanks for considering our revised manuscript. Please let us know if there is anything further needed at this stage. We will look forward to hearing from you.

Yours sincerely

Jane Alsweiler

j.alsweiler@auckland.ac.nz

1. Please ensure that your manuscript meets PLOS ONE's style requirements, including those for file naming. The PLOS ONE style templates can be found at 
[https://journals.plos.org/plosone/s/file?id=wjVg/PLOSOne_formatting_sample_main_body.pdf](https://protect-au.mimecast.com/s/0-jRC4QOrYIj1w2vCWbmxk?domain=journals.plos.org) and 
[https://journals.plos.org/plosone/s/file?id=ba62/PLOSOne_formatting_sample_title_authors_affiliations.pdf](https://protect-au.mimecast.com/s/jURdC5QPv1InGrPDSxNSga?domain=journals.plos.org)

*We have ensured that the manuscript meets the style requirements*

2. You indicated that you had ethical approval for your study. In your Methods section, please ensure you have also stated whether you obtained consent from parents or guardians of the minors included in the study or whether the research ethics committee or IRB specifically waived the need for their consent.

*This included in the second paragraph of the methods* “Consent by the parents of babies whose data was included in the trial was waived by the ethics committee.”

3. We note that the grant information you provided in the ‘Funding Information’ and ‘Financial Disclosure’ sections do not match.

When you resubmit, please ensure that you provide the correct grant numbers for the awards you received for your study in the ‘Funding Information’ section.

*Both the financial disclosure and the funding information correctly give the grant number as STF-14-04 and the granting body as Gravida: National Centre for Growth and Development. Is the discrepancy that in the disclosure the grant holders are listed as JA, CC and JH, which is correct, but the funding information only allows for the principal investigator, JA to be listed?*

4. In your Data Availability statement, you have not specified where the minimal data set underlying the results described in your manuscript can be found. PLOS defines a study's minimal data set as the underlying data used to reach the conclusions drawn in the manuscript and any additional data required to replicate the reported study findings in their entirety. All PLOS journals require that the minimal data set be made fully available. For more information about our data policy, please see [http://journals.plos.org/plosone/s/data-availability](https://protect-au.mimecast.com/s/NCZZC6XQwzS3K46BILuWjc?domain=journals.plos.org).

Upon re-submitting your revised manuscript, please upload your study’s minimal underlying data set as either Supporting Information files or to a stable, public repository and include the relevant URLs, DOIs, or accession numbers within your revised cover letter. For a list of acceptable repositories, please see [http://journals.plos.org/plosone/s/data-availability#loc-recommended-repositories](https://protect-au.mimecast.com/s/tIipC71RxWHPkBXJh0Odw3?domain=journals.plos.org). Any potentially identifying patient information must be fully anonymized.

Important: If there are ethical or legal restrictions to sharing your data publicly, please explain these restrictions in detail. Please see our guidelines for more information on what we consider unacceptable restrictions to publicly sharing data: [http://journals.plos.org/plosone/s/data-availability#loc-unacceptable-data-access-restrictions](https://protect-au.mimecast.com/s/fhxEC81VyBHJRy5KsR1tJ8?domain=journals.plos.org). Note that it is not acceptable for the authors to be the sole named individuals responsible for ensuring data access.

We will update your Data Availability statement to reflect the information you provide in your cover letter.

*The dataset has been uploaded to a public repository as requested (*Alsweiler, Jane (2022): DesIGN trial dataset and data dictionary. The University of Auckland. Dataset. <https://doi.org/10.17608/k6.auckland.20174231.v1>*). As participants did not provide consent for their data to be made available the data has been fully anonymised, including the removal of any potential identifiers including: date of birth (and date of blood glucose testing as this is usually the date of birth), hospital name, sex, anthropometry, ethnicity and multiples. [1]*

5. We note that you have included the phrase “data not shown” in your manuscript. Unfortunately, this does not meet our data sharing requirements. PLOS does not permit references to inaccessible data. We require that authors provide all relevant data within the paper, Supporting Information files, or in an acceptable, public repository. Please add a citation to support this phrase or upload the data that corresponds with these findings to a stable repository (such as Figshare or Dryad) and provide and URLs, DOIs, or accession numbers that may be used to access these data. Or, if the data are not a core part of the research being presented in your study, we ask that you remove the phrase that refers to these data.

*The data has been uploaded to a repository, and this phrase has been removed.*

Additional Editor Comments:

Editor’s comments
1. The authors have mentioned several times in the manuscript that steps were taken to ensure that clinicians in the participating hospitals were unaware of the trial. The reason given was that clinicians and midwives may “compete” and this may make them perform far better than what they would under real-life conditions. What is not clear from the manuscript is:
a. Were midwives aware of the trial? Everywhere, the authors have only mentioned that CLINICIANS were not aware of the trial. If midwives were aware, wouldn’t they have an urge to compete with the doctors, thus inflating the differences? The hypothesis of the authors was that midwives were more effective leaders for implementing this guideline. If midwives were aware of the trial, but clinicians were not, it would introduce a bias.

*By clinicians we mean both midwives and doctors, we have clarified this in the manuscript line 98. Both midwives and doctors were unaware of the trial.*

b. The protocol of the trial had already been published. Did the author seriously believe that for a two-year period from 2015-2017 the clinicians in the participating centres would remain unaware that a trial was being conducted?

*The implementation of the guideline was completed by May 2017, and we purposefully did not publish the protocol of the trial until November 2017 to keep clinicians unaware of the trial during implementation. The only people on the country who were aware of the trial were the Steering group (3 people), the trial co-ordinator, the central research doctor and midwife, the funding body committee, the ethics committee, and the CEO of each hospital, all of whom were bound to keep the trial confidential. The trial was registered with the ISRCTN, rather than the local Australasian clinical trial registry (ANZCTR). We have recently published a description of the significant efforts that we went to in order to keep clinicians unaware of the trial.[2] New Zealand is a small country, with an active perinatal network of doctors and midwives of which the authors are an integral part. We remain certain that clinicians were unaware of the trial during implementation of the guideline.*

c. It appears that a research nurse and research doctor was recruited at each centre, who was not a part of the existing staff. Wouldn’t the presence of extra research staff on the premises and their involvement in identifying team leaders “give away” the fact that a clinical trial was underway.

*Our apologies that this was not clear in the manuscript. There was only one research midwife and one research doctor who were employed centrally at the university, not at a hospital. Their role was to contact the charge midwife or clinical director respectively at each hospital and ask them to appoint a local midwife or doctor to lead the implementation. The hospital staff were told that we were collecting data on the use of dextrose gel as part of an audit of the implementation, and the data collection was done by a local research nurse or midwife. We have clarified this in the manuscript lines 120-123.*

d. Couldn’t information from the midwives (who were presumably aware of the trial) not have easily leaked out to the other participating centres?

*There was only one midwife in the country who was aware of the trial (the central research midwife) and she was under strict instructions to keep the study confidential.*

e. At the end of the trial, did the investigators survey the clinicians to check whether they knew that a trial had been conducted?

*We did not survey the clinicians at the end of the study to check if they knew a trial had been conducted. In retrospect this would have been a good idea. However, as discussed above, we remain confident that clinicians were not aware the trial was being conducted during implementation of the guideline.*

2. What is the meaning of the statement “consent by the parents of babies WHOSE DATA WAS INCLUDED in the trial was waived by the ethics committee”? Since data from 6 centres was not included in the trial does it mean consent was waived for 15 centres whose data was finally included, but not waived for 6 centres whose data was not included?

*The need for individual consent by parents was waived at all centres. This sentence has been clarified, line 102*

3. Why did the research midwife require prior research experience in the prevention of neonatal hypoglycemia? The research midwife’s job was to identify another key midwife at the hospital to lead the implementation. Unless the research midwife was herself involved in patient management?

*The research midwife role did not require prior research experience in the prevention of neonatal hypoglycaemia, but the person in this role did have this experience so we included the information, as we included the research experience of the research doctor. We agree that this information is not relevant, and it has been removed.*

*The research midwife had to contact the charge midwife at multiple different hospitals and ask the charge midwife to identify a midwife to lead the implementation. This was clear in the published protocol, and our apologies for not making this clear in the manuscript. We have clarified this point, thank you for pointing this out.* *Lines 120-123.*

4. On what basis did the authors assume an ICC of 0.05 during sample size calculation?

*There were no available data on which to calculate the ICC, so a pragmatic assumption was made using 0.05 as the mid-point of commonly assumed ICC calculations. [3]*

5. The sample size calculation is based upon a single point comparison between the proportion of eligible babies treated with dextrose gel at 3 months. The assumption is that in the control arm (doctors), the proportion treated would be 40% and in the intervention arm (midwives) it would be 60%. I cross-checked it on an online calculator ([https://clusterrcts.shinyapps.io/rshinyapp/](https://protect-au.mimecast.com/s/Zm2QC91WzLHZBwWpHGHYxk?domain=clusterrcts.shinyapps.io)). The sample size of 20 clusters of 20 each is correct AS LONG AS it is assumed to be a parallel design WITH NO COMPARISON WITH BASELINE MEASUREMENTS, with a cross-sectional sampling structure (ie. a different set of patients measured at different time points as in this study), and the ICC assumed to be constant across time points (as in the study). The authors have not explicitly stated any of these assumptions in the manuscript, and one is forced to read them between the lines.

*The reviewer is correct that the sample size calculation was based on a parallel cluster randomised trial design (20 hospitals, 20 babies per hospital) with the primary outcome at 3 months, and did not take into consideration the comparison with the baseline measurements.*

6. Contrary to the sample size calculation, the study is actually analyzed as a change in the proportion of subjects treated with dextrose gel from baseline measurement to 3 months post-implementation. Not just that, the average of the change in proportions from various clusters within each arm are converted into a mean (standard deviation), and it is the difference between the means that is finally projected as the primary outcome. This is quite different from the original primary outcome mentioned in the methods. At the analysis stage, the authors have converted the primary outcome into a continuous outcome based upon the difference between a baseline measurement and a post-implementation measurement. The sample size would have been different had the authors taken this approach from the beginning.

*The reviewer is correct about the inconsistency between the initial sample size calculation (using a clustered design at both hospital and baby levels) and the stated primary outcome in the published study protocol (which was defined at the hospital level only). The following outcome analysis was agreed in the Statistical Analysis Plan to accommodate the inconsistency:*

*“The primary outcome (the change in proportion of eligible babies treated with dextrose gel) is defined at the hospital level, which will be summarised descriptively at each hospital and compared between two randomised groups at 3 and 6 months after implementation using linear mixed regression models. The primary analysis will adjust for the type of maternity hospital (primary, secondary, tertiary) and current use of oral dextrose gel to treat hypoglycaemic babies (yes, no) as stratification factors for randomisation. Repeated measures at the hospital level will be taken into account using a random cluster effect. The effect of intervention will be evaluated at each visit using an interaction term between randomised groups and assessment periods. Model-adjusted mean difference between two groups will be estimated with 95% confidence interval and two-sided p-value.*

*Note that the above analyses conducted at the hospital level are considered most appropriate for the defined study outcomes presented in the published study protocol. For a cluster randomised trial, however, it is more efficient to use all data collected at hospital and individual levels including all eligible babies recruited at baseline, 3 months and 6 months after implementation. Generalised linear mixed models with a logit link for Binomial distribution will be used to estimate the difference in proportion of eligible babies treated with dextrose gel between randomisation groups in each period, as well as the change between the 3 periods. The primary analysis will adjusted for stratification factors (type of maternity hospital and current use of oral dextrose gel before implementation), and account for the random cluster effect of hospital. Adjusted odds ratio (OR) will be presented with 95% confidence interval and two-sided p-value. A significant OR of >1 indicates a positive effect of implementation, with a higher likelihood of treating eligible babies with dextrose gel at the hospital after implementation. The intra-cluster correlation coefficient will also be estimated using the between and within cluster variances estimated from the mixed model.”*

*The manuscript has been modified to make the two analyses clearer, lines 200-201 and 206-207, lines 316-7. In addition we have discussed the potential for Type 2 error in the Discussion lines 383-390.*

a. Why was the primary outcome not simply analyzed as a difference in the proportions at 3 months post-implementation, as was originally planned?

*It was always planned that the primary outcome would be analysed as the change in proportion at 3 months post implementation (not as a difference in the proportion at 3 months post-implementation), this was documented in both the published protocol and the trial registration. There was assumed to be a large difference in dextrose gel use in different maternity hospitals in New Zealand, with some hospitals not using any dextrose gel, and others routinely using dextrose gel to treat hypoglycaemic babies (such as the hospital involved in the original RCT). While the randomisation was stratified based on dextrose gel use, we believed that an analysis of change in dextrose gel use from baseline would be more appropriate than measuring a difference in proportions at 3 months, which would be heavily influenced by prior gel use.*

*We performed analysis on the primary outcome using both linear regression on change in proportion of babies treated with gel at 3 months only, and the mixed models on change in proportion of babies treated with gel at 3 and 6 months, adding an interaction term between randomised groups and assessment periods. The results of both analyses were consistent.*

b. The mean (standard deviation) of the change in proportions within each arm is based on just 7 to 8 data points, with each data point being contributed by one cluster. This is a matter of concern as mean and standard deviation is not meaningful with so few data points.

*We agree that the number of data points at the hospital level are small and have included descriptive summaries with median (IQR).*

7. Also, the implication of the sample size calculation was that there would be 400 subjects available for measuring the primary outcome at 3 months post-implementation. However, the authors have taken the sum of subjects available at baseline, at 3 months and at 6 months to be equal to the sample size. This is not the correct approach. Having only 163 subjects available at 3 months post-implementation makes the study underpowered.

*We agree that we were unable to achieve the planned sample size and this has been added as a limitation, line 380-387*

8. The authors have not provided data about the characteristics of the infants at each of the 3 time points (table 1 shows the aggregate data across the 3 time points).

*Table 1 has been adjusted to show the characteristics across each of the 3 time points.*

9. The authors have not provided the number of neonates available within each hospital at each of the time points. A wide variation in the cluster size at varying time points has implications for the power calculation and analysis.

*It is unclear what the reviewer means by the “number of neonates available within each hospital at each of the time points”. All eligible neonates were included in the study, so the number of neonates included is the number of neonates available. If the reviewer means the number of neonates born at each hospital at each timepoint, it is not relevant to include the number of neonates that were born but not eligible to be included in the trial.*

10. What were the reasons why as many as 6 out of 21 randomized hospitals failed to record data at both the time points? The figure mentions 1 centre did not collect data and 5 centres had too few eligible babies. Can the authors provide some more details?

*At each centre a research nurse or midwife collected the data, in one centre the research nurse did not complete the data collection and we were unable to recruit another local research nurse to do the data collection.*

*To calculate the primary outcome the change in the proportion of babies eligible to receive dextrose gel who were treated with dextrose gel from before implementation to 3 months after implementation, there was a requirement for each centre to have eligible babies i.e. babies with hypoglycaemia, both pre implementation and post implementation. We specified in the Statistical Analysis Plan that “If a hospital has no eligible babies during any of the 4 week periods this data point will be treated as missing.” We have clarified this in the paper.*

11. A 30% reduction in the number of clusters additionally contributes to a different sample size requirement.

*We agree and this has been added to the limitations, lines 380-387.*

12. The problem is further compounded by a highly unequal distribution of number of subjects randomized to the 2 arms by chance. The claim made in the discussion section (lines 402-404) that “the difference in size between the groups was present both before and after implementation of the guidelines and is unlikely to have change the results” is overly simplistic. Beyond a point, an unequal distribution between the 2 arms does result in a loss of power.

*We have reworded the limitation that we may not have had sufficient power to detect a difference between the groups.*

13. In the discussion section (line 382), the authors state that 80% of eligible babies were already treated with dextrose gel prior to implementation of the guideline. But table 2 mentions that pre-implementation, the proportion treated with dextrose gel was 70.7% and 63.3% in the 2 groups respectively. Not clear from where the figure of 80% came?

*In Line 297 of the results we present the data: Overall, there was an increase in the proportion of eligible babies treated with oral dextrose gel from prior to implementation to 3 months after implementation (122/153 (80%) v 144/163 (88%), OR( 95%CI); 3.42 (1.67-6.98), p<0.001).*

*In Table 2 the pre-implementation mean proportions of hypoglycaemic babies at each hospital, presented as mean (SD) are 70.7% and 63.3% for the two groups.*

*This difference reflects that the mean of a group of proportions is not the same as the proportion of the whole cohort.*

1.	Hrynaszkiewicz I, Norton ML, Vickers AJ, Altman DG. Preparing raw clinical data for publication: guidance for journal editors, authors, and peer reviewers. 2010;340:c181.

2.	Kuo J, Woodall S, Harding J, Crowther C, Alsweiler J. The challenges of keeping clinicians unaware of their participation in a national, cluster-randomised, implementation trial. BMC Med Ethics. 2022;23(1):55.

3.	Hemming K, Taljaard M. Sample size calculations for stepped wedge and cluster randomised trials: a unified approach. J Clin Epidemiol. 2016;69:137-46.
