# Supplementary material for: Midwife or doctor leader to implement a national guideline in babies on postnatal wards (DesIGN): A cluster-randomised, controlled, trial
Source: PLoS One. 2023 Sep 28;18(9):e0291784. doi: 10.1371/journal.pone.0291784 (PMC10538667; doi:10.1371/journal.pone.0291784)
Supplement: Attachment — Submitted filename: Response to Reviewers.docx [file pone.0291784.s004.docx]

5^th^ October 2022

Response to Reviewers

1- Line 196: The standard terminology is “linear mixed regression model.”

Thank you for spotting this error, which has been corrected.

2- State the underlying covariance structure used in the linear mixed regression model and the criteria for selecting it.

This has been added, lines 202-204.

3- Line 225: Clarify the statement, “A sensitivity analysis was conducted excluding multiples from the analysis.”

This has been clarified, lines 228-229

4- Define the abbreviations SD and IQR at their first occurrence.

These have been defined.

5- Improve the clarity of table 1 by indicating that the lines with bolded text represent the sample sizes (n).

Thank you, this has been clarified.

6- Line 301: Provide a more precise p-value instead of p< 0.05.

This p value has been included.
