# Supplementary material for: Midwife or doctor leader to implement a national guideline in babies on postnatal wards (DesIGN): A cluster-randomised, controlled, trial
Source: PLoS One. 2023 Sep 28;18(9):e0291784. doi: 10.1371/journal.pone.0291784 (PMC10538667; doi:10.1371/journal.pone.0291784)
Supplement: Peer review history

# Editor report: Decision Letter 0

**DOI:** 10.1371/journal.pone.0291784.r001

**Authors:** Sourabh Dutta

11 May 2022

PONE-D-22-10106Midwife or doctor leader to implement a national guideline in babies on postnatal wards (DesIGN): a cluster-randomised, controlled, trial.PLOS ONE

Dear Dr. Alsweiler,

Thank you for submitting your manuscript to PLOS ONE. After careful consideration, we feel that it has merit but does not fully meet PLOS ONE’s publication criteria as it currently stands. Therefore, we invite you to submit a revised version of the manuscript that addresses the points raised during the review process.

The points raised are mentioned below under "Editor's comments".

Please submit your revised manuscript by Jun 25 2022 11:59PM. If you will need more time than this to complete your revisions, please reply to this message or contact the journal office at plosone@plos.org. When you're ready to submit your revision, log on to https://www.editorialmanager.com/pone/ and select the 'Submissions Needing Revision' folder to locate your manuscript file.

Please include the following items when submitting your revised manuscript:

- A rebuttal letter that responds to each point raised by the academic editor and reviewer(s). You should upload this letter as a separate file labeled 'Response to Reviewers'.
- A marked-up copy of your manuscript that highlights changes made to the original version. You should upload this as a separate file labeled 'Revised Manuscript with Track Changes'.
- An unmarked version of your revised paper without tracked changes. You should upload this as a separate file labeled 'Manuscript'.

If you would like to make changes to your financial disclosure, please include your updated statement in your cover letter. Guidelines for resubmitting your figure files are available below the reviewer comments at the end of this letter.

If applicable, we recommend that you deposit your laboratory protocols in protocols.io to enhance the reproducibility of your results. Protocols.io assigns your protocol its own identifier (DOI) so that it can be cited independently in the future. For instructions see: https://journals.plos.org/plosone/s/submission-guidelines#loc-laboratory-protocols. Additionally, PLOS ONE offers an option for publishing peer-reviewed Lab Protocol articles, which describe protocols hosted on protocols.io. Read more information on sharing protocols at https://plos.org/protocols?utm_medium=editorial-email&utm_source=authorletters&utm_campaign=protocols.

We look forward to receiving your revised manuscript.

Kind regards,

Sourabh Dutta

Academic Editor

PLOS ONE

Journal Requirements:

When submitting your revision, we need you to address these additional requirements.

1. Please ensure that your manuscript meets PLOS ONE's style requirements, including those for file naming. The PLOS ONE style templates can be found at 

https://journals.plos.org/plosone/s/file?id=wjVg/PLOSOne_formatting_sample_main_body.pdf and 

https://journals.plos.org/plosone/s/file?id=ba62/PLOSOne_formatting_sample_title_authors_affiliations.pdf

2. You indicated that you had ethical approval for your study. In your Methods section, please ensure you have also stated whether you obtained consent from parents or guardians of the minors included in the study or whether the research ethics committee or IRB specifically waived the need for their consent.

3. We note that the grant information you provided in the ‘Funding Information’ and ‘Financial Disclosure’ sections do not match. 

When you resubmit, please ensure that you provide the correct grant numbers for the awards you received for your study in the ‘Funding Information’ section.

4. In your Data Availability statement, you have not specified where the minimal data set underlying the results described in your manuscript can be found. PLOS defines a study's minimal data set as the underlying data used to reach the conclusions drawn in the manuscript and any additional data required to replicate the reported study findings in their entirety. All PLOS journals require that the minimal data set be made fully available. For more information about our data policy, please see http://journals.plos.org/plosone/s/data-availability.

Upon re-submitting your revised manuscript, please upload your study’s minimal underlying data set as either Supporting Information files or to a stable, public repository and include the relevant URLs, DOIs, or accession numbers within your revised cover letter. For a list of acceptable repositories, please see http://journals.plos.org/plosone/s/data-availability#loc-recommended-repositories. Any potentially identifying patient information must be fully anonymized.

Important: If there are ethical or legal restrictions to sharing your data publicly, please explain these restrictions in detail. Please see our guidelines for more information on what we consider unacceptable restrictions to publicly sharing data: http://journals.plos.org/plosone/s/data-availability#loc-unacceptable-data-access-restrictions. Note that it is not acceptable for the authors to be the sole named individuals responsible for ensuring data access.

We will update your Data Availability statement to reflect the information you provide in your cover letter.

5. We note that you have included the phrase “data not shown” in your manuscript. Unfortunately, this does not meet our data sharing requirements. PLOS does not permit references to inaccessible data. We require that authors provide all relevant data within the paper, Supporting Information files, or in an acceptable, public repository. Please add a citation to support this phrase or upload the data that corresponds with these findings to a stable repository (such as Figshare or Dryad) and provide and URLs, DOIs, or accession numbers that may be used to access these data. Or, if the data are not a core part of the research being presented in your study, we ask that you remove the phrase that refers to these data.

Additional Editor Comments:

Editor’s comments

1. The authors have mentioned several times in the manuscript that steps were taken to ensure that clinicians in the participating hospitals were unaware of the trial. The reason given was that clinicians and midwives may “compete” and this may make them perform far better than what they would under real-life conditions. What is not clear from the manuscript is:

a. Were midwives aware of the trial? Everywhere, the authors have only mentioned that CLINICIANS were not aware of the trial. If midwives were aware, wouldn’t they have an urge to compete with the doctors, thus inflating the differences? The hypothesis of the authors was that midwives were more effective leaders for implementing this guideline. If midwives were aware of the trial, but clinicians were not, it would introduce a bias.

b. The protocol of the trial had already been published. Did the author seriously believe that for a two-year period from 2015-2017 the clinicians in the participating centres would remain unaware that a trial was being conducted?

c. It appears that a research nurse and research doctor was recruited at each centre, who was not a part of the existing staff. Wouldn’t the presence of extra research staff on the premises and their involvement in identifying team leaders “give away” the fact that a clinical trial was underway.

d. Couldn’t information from the midwives (who were presumably aware of the trial) not have easily leaked out to the other participating centres?

e. At the end of the trial, did the investigators survey the clinicians to check whether they knew that a trial had been conducted?

2. What is the meaning of the statement “consent by the parents of babies WHOSE DATA WAS INCLUDED in the trial was waived by the ethics committee”? Since data from 6 centres was not included in the trial does it mean consent was waived for 15 centres whose data was finally included, but not waived for 6 centres whose data was not included?

3. Why did the research midwife require prior research experience in the prevention of neonatal hypoglycemia? The research midwife’s job was to identify another key midwife at the hospital to lead the implementation. Unless the research midwife was herself involved in patient management?

4. On what basis did the authors assume an ICC of 0.05 during sample size calculation?

5. The sample size calculation is based upon a single point comparison between the proportion of eligible babies treated with dextrose gel at 3 months. The assumption is that in the control arm (doctors), the proportion treated would be 40% and in the intervention arm (midwives) it would be 60%. I cross-checked it on an online calculator (https://clusterrcts.shinyapps.io/rshinyapp/). The sample size of 20 clusters of 20 each is correct AS LONG AS it is assumed to be a parallel design WITH NO COMPARISON WITH BASELINE MEASUREMENTS, with a cross-sectional sampling structure (ie. a different set of patients measured at different time points as in this study), and the ICC assumed to be constant across time points (as in the study). The authors have not explicitly stated any of these assumptions in the manuscript, and one is forced to read them between the lines.

6. Contrary to the sample size calculation, the study is actually analyzed as a change in the proportion of subjects treated with dextrose gel from baseline measurement to 3 months post-implementation. Not just that, the average of the change in proportions from various clusters within each arm are converted into a mean (standard deviation), and it is the difference between the means that is finally projected as the primary outcome. This is quite different from the original primary outcome mentioned in the methods. At the analysis stage, the authors have converted the primary outcome into a continuous outcome based upon the difference between a baseline measurement and a post-implementation measurement. The sample size would have been different had the authors taken this approach from the beginning.

a. Why was the primary outcome not simply analyzed as a difference in the proportions at 3 months post-implementation, as was originally planned?

b. The mean (standard deviation) of the change in proportions within each arm is based on just 7 to 8 data points, with each data point being contributed by one cluster. This is a matter of concern as mean and standard deviation is not meaningful with so few data points.

7. Also, the implication of the sample size calculation was that there would be 400 subjects available for measuring the primary outcome at 3 months post-implementation. However, the authors have taken the sum of subjects available at baseline, at 3 months and at 6 months to be equal to the sample size. This is not the correct approach. Having only 163 subjects available at 3 months post-implementation makes the study underpowered.

8. The authors have not provided data about the characteristics of the infants at each of the 3 time points (table 1 shows the aggregate data across the 3 time points).

9. The authors have not provided the number of neonates available within each hospital at each of the time points. A wide variation in the cluster size at varying time points has implications for the power calculation and analysis.

10. What were the reasons why as many as 6 out of 21 randomized hospitals failed to record data at both the time points? The figure mentions 1 centre did not collect data and 5 centres had too few eligible babies. Can the authors provide some more details?

11. A 30% reduction in the number of clusters additionally contributes to a different sample size requirement.

12. The problem is further compounded by a highly unequal distribution of number of subjects randomized to the 2 arms by chance. The claim made in the discussion section (lines 402-404) that “the difference in size between the groups was present both before and after implementation of the guidelines and is unlikely to have change the results” is overly simplistic. Beyond a point, an unequal distribution between the 2 arms does result in a loss of power.

13. In the discussion section (line 382), the authors state that 80% of eligible babies were already treated with dextrose gel prior to implementation of the guideline. But table 2 mentions that pre-implementation, the proportion treated with dextrose gel was 70.7% and 63.3% in the 2 groups respectively. Not clear from where the figure of 80% came?

14. This was planned as a superiority, cluster-randomized controlled trial. In any superiority trial, inability to detect a statistically significant difference does not imply equivalence or noninferiority. Some of the sentences used by the authors towards the end of the discussion and in the conclusion imply that doctors and midwives were considered equally effective at implementing clinical practice guidelines. However, this conclusion cannot be drawn. All that one can say is the authors failed to show a difference between doctor-led and midwife-led implementation, but not that midwives are as effective as doctors in implementing the guidelines.

[Note: HTML markup is below. Please do not edit.]

Reviewers' comments:

[NOTE: If reviewer comments were submitted as an attachment file, they will be attached to this email and accessible via the submission site. Please log into your account, locate the manuscript record, and check for the action link "View Attachments". If this link does not appear, there are no attachment files.]

While revising your submission, please upload your figure files to the Preflight Analysis and Conversion Engine (PACE) digital diagnostic tool, https://pacev2.apexcovantage.com/. PACE helps ensure that figures meet PLOS requirements. To use PACE, you must first register as a user. Registration is free. Then, login and navigate to the UPLOAD tab, where you will find detailed instructions on how to use the tool. If you encounter any issues or have any questions when using PACE, please email PLOS at figures@plos.org. Please note that Supporting Information files do not need this step.

# Author comment: Author response to Decision Letter 0

**DOI:** 10.1371/journal.pone.0291784.r002

29 Jun 2022

1. Please ensure that your manuscript meets PLOS ONE's style requirements, including those for file naming. The PLOS ONE style templates can be found at 

https://journals.plos.org/plosone/s/file?id=wjVg/PLOSOne_formatting_sample_main_body.pdf and 

https://journals.plos.org/plosone/s/file?id=ba62/PLOSOne_formatting_sample_title_authors_affiliations.pdf

We have ensured that the manuscript meets the style requirements

2. You indicated that you had ethical approval for your study. In your Methods section, please ensure you have also stated whether you obtained consent from parents or guardians of the minors included in the study or whether the research ethics committee or IRB specifically waived the need for their consent.

This included in the second paragraph of the methods “Consent by the parents of babies whose data was included in the trial was waived by the ethics committee.”

3. We note that the grant information you provided in the ‘Funding Information’ and ‘Financial Disclosure’ sections do not match. 

When you resubmit, please ensure that you provide the correct grant numbers for the awards you received for your study in the ‘Funding Information’ section.

Both the financial disclosure and the funding information correctly give the grant number as STF-14-04 and the granting body as Gravida: National Centre for Growth and Development. Is the discrepancy that in the disclosure the grant holders are listed as JA, CC and JH, which is correct, but the funding information only allows for the principal investigator, JA to be listed?

4. In your Data Availability statement, you have not specified where the minimal data set underlying the results described in your manuscript can be found. PLOS defines a study's minimal data set as the underlying data used to reach the conclusions drawn in the manuscript and any additional data required to replicate the reported study findings in their entirety. All PLOS journals require that the minimal data set be made fully available. For more information about our data policy, please see http://journals.plos.org/plosone/s/data-availability.

Upon re-submitting your revised manuscript, please upload your study’s minimal underlying data set as either Supporting Information files or to a stable, public repository and include the relevant URLs, DOIs, or accession numbers within your revised cover letter. For a list of acceptable repositories, please see http://journals.plos.org/plosone/s/data-availability#loc-recommended-repositories. Any potentially identifying patient information must be fully anonymized.

Important: If there are ethical or legal restrictions to sharing your data publicly, please explain these restrictions in detail. Please see our guidelines for more information on what we consider unacceptable restrictions to publicly sharing data: http://journals.plos.org/plosone/s/data-availability#loc-unacceptable-data-access-restrictions. Note that it is not acceptable for the authors to be the sole named individuals responsible for ensuring data access.

We will update your Data Availability statement to reflect the information you provide in your cover letter.

The dataset has been uploaded to a public repository as requested (Alsweiler, Jane (2022): DesIGN trial dataset and data dictionary. The University of Auckland. Dataset. https://doi.org/10.17608/k6.auckland.20174231.v1). As participants did not provide consent for their data to be made available the data has been fully anonymised, including the removal of any potential identifiers including: date of birth (and date of blood glucose testing as this is usually the date of birth), hospital name, sex, anthropometry, ethnicity and multiples. [1]

5. We note that you have included the phrase “data not shown” in your manuscript. Unfortunately, this does not meet our data sharing requirements. PLOS does not permit references to inaccessible data. We require that authors provide all relevant data within the paper, Supporting Information files, or in an acceptable, public repository. Please add a citation to support this phrase or upload the data that corresponds with these findings to a stable repository (such as Figshare or Dryad) and provide and URLs, DOIs, or accession numbers that may be used to access these data. Or, if the data are not a core part of the research being presented in your study, we ask that you remove the phrase that refers to these data.

The data has been uploaded to a repository, and this phrase has been removed. 

Additional Editor Comments:

Editor’s comments

1. The authors have mentioned several times in the manuscript that steps were taken to ensure that clinicians in the participating hospitals were unaware of the trial. The reason given was that clinicians and midwives may “compete” and this may make them perform far better than what they would under real-life conditions. What is not clear from the manuscript is:

a. Were midwives aware of the trial? Everywhere, the authors have only mentioned that CLINICIANS were not aware of the trial. If midwives were aware, wouldn’t they have an urge to compete with the doctors, thus inflating the differences? The hypothesis of the authors was that midwives were more effective leaders for implementing this guideline. If midwives were aware of the trial, but clinicians were not, it would introduce a bias.

By clinicians we mean both midwives and doctors, we have clarified this in the manuscript line 98. Both midwives and doctors were unaware of the trial. 

b. The protocol of the trial had already been published. Did the author seriously believe that for a two-year period from 2015-2017 the clinicians in the participating centres would remain unaware that a trial was being conducted?

The implementation of the guideline was completed by May 2017, and we purposefully did not publish the protocol of the trial until November 2017 to keep clinicians unaware of the trial during implementation. The only people on the country who were aware of the trial were the Steering group (3 people), the trial co-ordinator, the central research doctor and midwife, the funding body committee, the ethics committee, and the CEO of each hospital, all of whom were bound to keep the trial confidential. The trial was registered with the ISRCTN, rather than the local Australasian clinical trial registry (ANZCTR). We have recently published a description of the significant efforts that we went to in order to keep clinicians unaware of the trial.[2] New Zealand is a small country, with an active perinatal network of doctors and midwives of which the authors are an integral part. We remain certain that clinicians were unaware of the trial during implementation of the guideline.

c. It appears that a research nurse and research doctor was recruited at each centre, who was not a part of the existing staff. Wouldn’t the presence of extra research staff on the premises and their involvement in identifying team leaders “give away” the fact that a clinical trial was underway.

Our apologies that this was not clear in the manuscript. There was only one research midwife and one research doctor who were employed centrally at the university, not at a hospital. Their role was to contact the charge midwife or clinical director respectively at each hospital and ask them to appoint a local midwife or doctor to lead the implementation. The hospital staff were told that we were collecting data on the use of dextrose gel as part of an audit of the implementation, and the data collection was done by a local research nurse or midwife. We have clarified this in the manuscript lines 120-123. 

d. Couldn’t information from the midwives (who were presumably aware of the trial) not have easily leaked out to the other participating centres?

There was only one midwife in the country who was aware of the trial (the central research midwife) and she was under strict instructions to keep the study confidential. 

e. At the end of the trial, did the investigators survey the clinicians to check whether they knew that a trial had been conducted?

We did not survey the clinicians at the end of the study to check if they knew a trial had been conducted. In retrospect this would have been a good idea. However, as discussed above, we remain confident that clinicians were not aware the trial was being conducted during implementation of the guideline. 

2. What is the meaning of the statement “consent by the parents of babies WHOSE DATA WAS INCLUDED in the trial was waived by the ethics committee”? Since data from 6 centres was not included in the trial does it mean consent was waived for 15 centres whose data was finally included, but not waived for 6 centres whose data was not included?

The need for individual consent by parents was waived at all centres. This sentence has been clarified, line 102

3. Why did the research midwife require prior research experience in the prevention of neonatal hypoglycemia? The research midwife’s job was to identify another key midwife at the hospital to lead the implementation. Unless the research midwife was herself involved in patient management?

The research midwife role did not require prior research experience in the prevention of neonatal hypoglycaemia, but the person in this role did have this experience so we included the information, as we included the research experience of the research doctor. We agree that this information is not relevant, and it has been removed. 

The research midwife had to contact the charge midwife at multiple different hospitals and ask the charge midwife to identify a midwife to lead the implementation. This was clear in the published protocol, and our apologies for not making this clear in the manuscript. We have clarified this point, thank you for pointing this out. Lines 120-123. 

4. On what basis did the authors assume an ICC of 0.05 during sample size calculation?

There were no available data on which to calculate the ICC, so a pragmatic assumption was made using 0.05 as the mid-point of commonly assumed ICC calculations. [3]

5. The sample size calculation is based upon a single point comparison between the proportion of eligible babies treated with dextrose gel at 3 months. The assumption is that in the control arm (doctors), the proportion treated would be 40% and in the intervention arm (midwives) it would be 60%. I cross-checked it on an online calculator (https://clusterrcts.shinyapps.io/rshinyapp/). The sample size of 20 clusters of 20 each is correct AS LONG AS it is assumed to be a parallel design WITH NO COMPARISON WITH BASELINE MEASUREMENTS, with a cross-sectional sampling structure (ie. a different set of patients measured at different time points as in this study), and the ICC assumed to be constant across time points (as in the study). The authors have not explicitly stated any of these assumptions in the manuscript, and one is forced to read them between the lines.

The reviewer is correct that the sample size calculation was based on a parallel cluster randomised trial design (20 hospitals, 20 babies per hospital) with the primary outcome at 3 months, and did not take into consideration the comparison with the baseline measurements. 

6. Contrary to the sample size calculation, the study is actually analyzed as a change in the proportion of subjects treated with dextrose gel from baseline measurement to 3 months post-implementation. Not just that, the average of the change in proportions from various clusters within each arm are converted into a mean (standard deviation), and it is the difference between the means that is finally projected as the primary outcome. This is quite different from the original primary outcome mentioned in the methods. At the analysis stage, the authors have converted the primary outcome into a continuous outcome based upon the difference between a baseline measurement and a post-implementation measurement. The sample size would have been different had the authors taken this approach from the beginning.

The reviewer is correct about the inconsistency between the initial sample size calculation (using a clustered design at both hospital and baby levels) and the stated primary outcome in the published study protocol (which was defined at the hospital level only). The following outcome analysis was agreed in the Statistical Analysis Plan to accommodate the inconsistency: 

“The primary outcome (the change in proportion of eligible babies treated with dextrose gel) is defined at the hospital level, which will be summarised descriptively at each hospital and compared between two randomised groups at 3 and 6 months after implementation using linear mixed regression models. The primary analysis will adjust for the type of maternity hospital (primary, secondary, tertiary) and current use of oral dextrose gel to treat hypoglycaemic babies (yes, no) as stratification factors for randomisation. Repeated measures at the hospital level will be taken into account using a random cluster effect. The effect of intervention will be evaluated at each visit using an interaction term between randomised groups and assessment periods. Model-adjusted mean difference between two groups will be estimated with 95% confidence interval and two-sided p-value.

Note that the above analyses conducted at the hospital level are considered most appropriate for the defined study outcomes presented in the published study protocol. For a cluster randomised trial, however, it is more efficient to use all data collected at hospital and individual levels including all eligible babies recruited at baseline, 3 months and 6 months after implementation. Generalised linear mixed models with a logit link for Binomial distribution will be used to estimate the difference in proportion of eligible babies treated with dextrose gel between randomisation groups in each period, as well as the change between the 3 periods. The primary analysis will adjusted for stratification factors (type of maternity hospital and current use of oral dextrose gel before implementation), and account for the random cluster effect of hospital. Adjusted odds ratio (OR) will be presented with 95% confidence interval and two-sided p-value. A significant OR of >1 indicates a positive effect of implementation, with a higher likelihood of treating eligible babies with dextrose gel at the hospital after implementation. The intra-cluster correlation coefficient will also be estimated using the between and within cluster variances estimated from the mixed model.”

The manuscript has been modified to make the two analyses clearer, lines 200-201 and 206-207, lines 316-7. In addition we have discussed the potential for Type 2 error in the Discussion lines 383-390. 

a. Why was the primary outcome not simply analyzed as a difference in the proportions at 3 months post-implementation, as was originally planned?

It was always planned that the primary outcome would be analysed as the change in proportion at 3 months post implementation (not as a difference in the proportion at 3 months post-implementation), this was documented in both the published protocol and the trial registration. There was assumed to be a large difference in dextrose gel use in different maternity hospitals in New Zealand, with some hospitals not using any dextrose gel, and others routinely using dextrose gel to treat hypoglycaemic babies (such as the hospital involved in the original RCT). While the randomisation was stratified based on dextrose gel use, we believed that an analysis of change in dextrose gel use from baseline would be more appropriate than measuring a difference in proportions at 3 months, which would be heavily influenced by prior gel use. 

We performed analysis on the primary outcome using both linear regression on change in proportion of babies treated with gel at 3 months only, and the mixed models on change in proportion of babies treated with gel at 3 and 6 months, adding an interaction term between randomised groups and assessment periods. The results of both analyses were consistent. 

b. The mean (standard deviation) of the change in proportions within each arm is based on just 7 to 8 data points, with each data point being contributed by one cluster. This is a matter of concern as mean and standard deviation is not meaningful with so few data points.

We agree that the number of data points at the hospital level are small and have included descriptive summaries with median (IQR).

7. Also, the implication of the sample size calculation was that there would be 400 subjects available for measuring the primary outcome at 3 months post-implementation. However, the authors have taken the sum of subjects available at baseline, at 3 months and at 6 months to be equal to the sample size. This is not the correct approach. Having only 163 subjects available at 3 months post-implementation makes the study underpowered.

We agree that we were unable to achieve the planned sample size and this has been added as a limitation, line 380-387

8. The authors have not provided data about the characteristics of the infants at each of the 3 time points (table 1 shows the aggregate data across the 3 time points).

Table 1 has been adjusted to show the characteristics across each of the 3 time points. 

9. The authors have not provided the number of neonates available within each hospital at each of the time points. A wide variation in the cluster size at varying time points has implications for the power calculation and analysis.

It is unclear what the reviewer means by the “number of neonates available within each hospital at each of the time points”. All eligible neonates were included in the study, so the number of neonates included is the number of neonates available. If the reviewer means the number of neonates born at each hospital at each timepoint, it is not relevant to include the number of neonates that were born but not eligible to be included in the trial. 

10. What were the reasons why as many as 6 out of 21 randomized hospitals failed to record data at both the time points? The figure mentions 1 centre did not collect data and 5 centres had too few eligible babies. Can the authors provide some more details?

At each centre a research nurse or midwife collected the data, in one centre the research nurse did not complete the data collection and we were unable to recruit another local research nurse to do the data collection. 

To calculate the primary outcome the change in the proportion of babies eligible to receive dextrose gel who were treated with dextrose gel from before implementation to 3 months after implementation, there was a requirement for each centre to have eligible babies i.e. babies with hypoglycaemia, both pre implementation and post implementation. We specified in the Statistical Analysis Plan that “If a hospital has no eligible babies during any of the 4 week periods this data point will be treated as missing.” We have clarified this in the paper. 

11. A 30% reduction in the number of clusters additionally contributes to a different sample size requirement.

We agree and this has been added to the limitations, lines 380-387.

12. The problem is further compounded by a highly unequal distribution of number of subjects randomized to the 2 arms by chance. The claim made in the discussion section (lines 402-404) that “the difference in size between the groups was present both before and after implementation of the guidelines and is unlikely to have change the results” is overly simplistic. Beyond a point, an unequal distribution between the 2 arms does result in a loss of power.

We have reworded the limitation that we may not have had sufficient power to detect a difference between the groups. 

13. In the discussion section (line 382), the authors state that 80% of eligible babies were already treated with dextrose gel prior to implementation of the guideline. But table 2 mentions that pre-implementation, the proportion treated with dextrose gel was 70.7% and 63.3% in the 2 groups respectively. Not clear from where the figure of 80% came?

In Line 297 of the results we present the data: Overall, there was an increase in the proportion of eligible babies treated with oral dextrose gel from prior to implementation to 3 months after implementation (122/153 (80%) v 144/163 (88%), OR( 95%CI); 3.42 (1.67-6.98), p<0.001). 

In Table 2 the pre-implementation mean proportions of hypoglycaemic babies at each hospital, presented as mean (SD) are 70.7% and 63.3% for the two groups. 

This difference reflects that the mean of a group of proportions is not the same as the proportion of the whole cohort.

1. Hrynaszkiewicz I, Norton ML, Vickers AJ, Altman DG. Preparing raw clinical data for publication: guidance for journal editors, authors, and peer reviewers. 2010;340:c181.

2. Kuo J, Woodall S, Harding J, Crowther C, Alsweiler J. The challenges of keeping clinicians unaware of their participation in a national, cluster-randomised, implementation trial. BMC Med Ethics. 2022;23(1):55.

3. Hemming K, Taljaard M. Sample size calculations for stepped wedge and cluster randomised trials: a unified approach. J Clin Epidemiol. 2016;69:137-46.

Attachment Submitted filename: Response to reviewers.docx Click here for additional data file.

# Peer review: Decision Letter 1

**DOI:** 10.1371/journal.pone.0291784.r003

**Authors:** Sourabh Dutta

19 Sep 2022

PONE-D-22-10106R1Midwife or doctor leader to implement a national guideline in babies on postnatal wards (DesIGN): a cluster-randomised, controlled, trial.PLOS ONE

Dear Dr. Alsweiler,

Thank you for submitting your manuscript to PLOS ONE. After careful consideration, we feel that it has merit but does not fully meet PLOS ONE’s publication criteria as it currently stands. Therefore, we invite you to submit a revised version of the manuscript that addresses the points raised during the review process.

==============================

A few minor, but important, changes have been suggested by Reviewer 1, which the authors must comply with. 

==============================

Please submit your revised manuscript by Nov 03 2022 11:59PM. If you will need more time than this to complete your revisions, please reply to this message or contact the journal office at plosone@plos.org. When you're ready to submit your revision, log on to https://www.editorialmanager.com/pone/ and select the 'Submissions Needing Revision' folder to locate your manuscript file.

Please include the following items when submitting your revised manuscript:

- A rebuttal letter that responds to each point raised by the academic editor and reviewer(s). You should upload this letter as a separate file labeled 'Response to Reviewers'.
- A marked-up copy of your manuscript that highlights changes made to the original version. You should upload this as a separate file labeled 'Revised Manuscript with Track Changes'.
- An unmarked version of your revised paper without tracked changes. You should upload this as a separate file labeled 'Manuscript'.

If you would like to make changes to your financial disclosure, please include your updated statement in your cover letter. Guidelines for resubmitting your figure files are available below the reviewer comments at the end of this letter.

If applicable, we recommend that you deposit your laboratory protocols in protocols.io to enhance the reproducibility of your results. Protocols.io assigns your protocol its own identifier (DOI) so that it can be cited independently in the future. For instructions see: https://journals.plos.org/plosone/s/submission-guidelines#loc-laboratory-protocols. Additionally, PLOS ONE offers an option for publishing peer-reviewed Lab Protocol articles, which describe protocols hosted on protocols.io. Read more information on sharing protocols at https://plos.org/protocols?utm_medium=editorial-email&utm_source=authorletters&utm_campaign=protocols.

We look forward to receiving your revised manuscript.

Kind regards,

Sourabh Dutta

Academic Editor

PLOS ONE

Journal Requirements:

Please review your reference list to ensure that it is complete and correct. If you have cited papers that have been retracted, please include the rationale for doing so in the manuscript text, or remove these references and replace them with relevant current references. Any changes to the reference list should be mentioned in the rebuttal letter that accompanies your revised manuscript. If you need to cite a retracted article, indicate the article’s retracted status in the References list and also include a citation and full reference for the retraction notice.

Additional Editor Comments:

The authors have already made major revisions to the manuscript with respect to the changes suggested by the academic reviewer. A few minor, but important, changes have been suggested by Reviewer 1, which the authors must comply with. 

[Note: HTML markup is below. Please do not edit.]

Reviewers' comments:

Reviewer's Responses to Questions

**Comments to the Author**

1. If the authors have adequately addressed your comments raised in a previous round of review and you feel that this manuscript is now acceptable for publication, you may indicate that here to bypass the “Comments to the Author” section, enter your conflict of interest statement in the “Confidential to Editor” section, and submit your "Accept" recommendation.

Reviewer #1: (No Response)

**********

2. Is the manuscript technically sound, and do the data support the conclusions?

The manuscript must describe a technically sound piece of scientific research with data that supports the conclusions. Experiments must have been conducted rigorously, with appropriate controls, replication, and sample sizes. The conclusions must be drawn appropriately based on the data presented. 

Reviewer #1: Yes

**********

3. Has the statistical analysis been performed appropriately and rigorously? 

Reviewer #1: Yes

**********

4. Have the authors made all data underlying the findings in their manuscript fully available?

The PLOS Data policy requires authors to make all data underlying the findings described in their manuscript fully available without restriction, with rare exception (please refer to the Data Availability Statement in the manuscript PDF file). The data should be provided as part of the manuscript or its supporting information, or deposited to a public repository. For example, in addition to summary statistics, the data points behind means, medians and variance measures should be available. If there are restrictions on publicly sharing data—e.g. participant privacy or use of data from a third party—those must be specified.

Reviewer #1: Yes

**********

5. Is the manuscript presented in an intelligible fashion and written in standard English?

PLOS ONE does not copyedit accepted manuscripts, so the language in submitted articles must be clear, correct, and unambiguous. Any typographical or grammatical errors should be corrected at revision, so please note any specific errors here.

Reviewer #1: Yes

**********

6. Review Comments to the Author

Please use the space provided to explain your answers to the questions above. You may also include additional comments for the author, including concerns about dual publication, research ethics, or publication ethics. (Please upload your review as an attachment if it exceeds 20,000 characters)

Reviewer #1: A multi-center, cluster-randomized controlled clinical trial was conducted which aimed to compare the effectiveness of midwives to doctor leaders at implementing a clinical practice guideline for oral dextrose gel to treat neonatal hypoglycemia. No differences in the change in proportion of hypoglycemic babies was observed between the two groups. An increase in the proportion of eligible babies treated with oral dextrose gel from pre-study to 3-months post implementation was observed.

Minor revisions:

1- Line 196: The standard terminology is “linear mixed regression model.”

2- State the underlying covariance structure used in the linear mixed regression model and the criteria for selecting it.

3- Line 225: Clarify the statement, “A sensitivity analysis was conducted excluding multiples from the analysis.”

4- Define the abbreviations SD and IQR at their first occurrence.

5- Improve the clarity of table 1 by indicating that the lines with bolded text represent the sample sizes (n).

6- Line 301: Provide a more precise p-value instead of p< 0.05.

**********

7. PLOS authors have the option to publish the peer review history of their article (what does this mean?). If published, this will include your full peer review and any attached files.

If you choose “no”, your identity will remain anonymous but your review may still be made public.

**Do you want your identity to be public for this peer review?** For information about this choice, including consent withdrawal, please see our Privacy Policy.

Reviewer #1: No

**********

[NOTE: If reviewer comments were submitted as an attachment file, they will be attached to this email and accessible via the submission site. Please log into your account, locate the manuscript record, and check for the action link "View Attachments". If this link does not appear, there are no attachment files.]

While revising your submission, please upload your figure files to the Preflight Analysis and Conversion Engine (PACE) digital diagnostic tool, https://pacev2.apexcovantage.com/. PACE helps ensure that figures meet PLOS requirements. To use PACE, you must first register as a user. Registration is free. Then, login and navigate to the UPLOAD tab, where you will find detailed instructions on how to use the tool. If you encounter any issues or have any questions when using PACE, please email PLOS at figures@plos.org. Please note that Supporting Information files do not need this step.

# Author comment: Author response to Decision Letter 1

**DOI:** 10.1371/journal.pone.0291784.r004

4 Oct 2022

1- Line 196: The standard terminology is “linear mixed regression model.”

Thank you for spotting this error, which has been corrected. 

2- State the underlying covariance structure used in the linear mixed regression model and the criteria for selecting it.

This has been added, lines 202-204.

3- Line 225: Clarify the statement, “A sensitivity analysis was conducted excluding multiples from the analysis.”

This has been clarified, lines 228-229

4- Define the abbreviations SD and IQR at their first occurrence.

These have been defined. 

5- Improve the clarity of table 1 by indicating that the lines with bolded text represent the sample sizes (n).

Thank you, this has been clarified. 

6- Line 301: Provide a more precise p-value instead of p< 0.05.

This p value has been included.

Attachment Submitted filename: Response to Reviewers.docx Click here for additional data file.

# Peer review: Decision Letter 2

**DOI:** 10.1371/journal.pone.0291784.r005

**Authors:** Sourabh Dutta

26 Jun 2023

PONE-D-22-10106R2

Midwife or doctor leader to implement a national guideline in babies on postnatal wards (DesIGN): a cluster-randomised, controlled, trial.

PLOS ONE

Dear Dr. Alsweiler,

Thank you for submitting your manuscript to PLOS ONE. After careful consideration, we feel that it has merit but does not fully meet PLOS ONE’s publication criteria as it currently stands. Therefore, we invite you to submit a revised version of the manuscript that addresses the points raised during the review process.

==============================

Kindly address the points raised by Reviewer 2.

==============================

Please submit your revised manuscript by Aug 10 2023 11:59PM. If you will need more time than this to complete your revisions, please reply to this message or contact the journal office at plosone@plos.org. When you're ready to submit your revision, log on to https://www.editorialmanager.com/pone/ and select the 'Submissions Needing Revision' folder to locate your manuscript file.

Please include the following items when submitting your revised manuscript:

- A rebuttal letter that responds to each point raised by the academic editor and reviewer(s). You should upload this letter as a separate file labeled 'Response to Reviewers'.
- A marked-up copy of your manuscript that highlights changes made to the original version. You should upload this as a separate file labeled 'Revised Manuscript with Track Changes'.
- An unmarked version of your revised paper without tracked changes. You should upload this as a separate file labeled 'Manuscript'.

If you would like to make changes to your financial disclosure, please include your updated statement in your cover letter. Guidelines for resubmitting your figure files are available below the reviewer comments at the end of this letter.

If applicable, we recommend that you deposit your laboratory protocols in protocols.io to enhance the reproducibility of your results. Protocols.io assigns your protocol its own identifier (DOI) so that it can be cited independently in the future. For instructions see: https://journals.plos.org/plosone/s/submission-guidelines#loc-laboratory-protocols. Additionally, PLOS ONE offers an option for publishing peer-reviewed Lab Protocol articles, which describe protocols hosted on protocols.io. Read more information on sharing protocols at https://plos.org/protocols?utm_medium=editorial-email&utm_source=authorletters&utm_campaign=protocols.

We look forward to receiving your revised manuscript.

Kind regards,

Sourabh Dutta

Academic Editor

PLOS ONE

Journal Requirements:

Please review your reference list to ensure that it is complete and correct. If you have cited papers that have been retracted, please include the rationale for doing so in the manuscript text, or remove these references and replace them with relevant current references. Any changes to the reference list should be mentioned in the rebuttal letter that accompanies your revised manuscript. If you need to cite a retracted article, indicate the article’s retracted status in the References list and also include a citation and full reference for the retraction notice.

Additional Editor Comments:

The authors have satisfactorily made the minor revisions as required. The manuscript is acceptable for publication in its current form.

[Note: HTML markup is below. Please do not edit.]

Reviewers' comments:

Reviewer's Responses to Questions

**Comments to the Author**

1. If the authors have adequately addressed your comments raised in a previous round of review and you feel that this manuscript is now acceptable for publication, you may indicate that here to bypass the “Comments to the Author” section, enter your conflict of interest statement in the “Confidential to Editor” section, and submit your "Accept" recommendation.

Reviewer #2: (No Response)

********** 

2. Is the manuscript technically sound, and do the data support the conclusions?

The manuscript must describe a technically sound piece of scientific research with data that supports the conclusions. Experiments must have been conducted rigorously, with appropriate controls, replication, and sample sizes. The conclusions must be drawn appropriately based on the data presented. 

Reviewer #2: Yes

********** 

3. Has the statistical analysis been performed appropriately and rigorously? 

Reviewer #2: Yes

********** 

4. Have the authors made all data underlying the findings in their manuscript fully available?

The PLOS Data policy requires authors to make all data underlying the findings described in their manuscript fully available without restriction, with rare exception (please refer to the Data Availability Statement in the manuscript PDF file). The data should be provided as part of the manuscript or its supporting information, or deposited to a public repository. For example, in addition to summary statistics, the data points behind means, medians and variance measures should be available. If there are restrictions on publicly sharing data—e.g. participant privacy or use of data from a third party—those must be specified.

Reviewer #2: Yes

********** 

5. Is the manuscript presented in an intelligible fashion and written in standard English?

PLOS ONE does not copyedit accepted manuscripts, so the language in submitted articles must be clear, correct, and unambiguous. Any typographical or grammatical errors should be corrected at revision, so please note any specific errors here.

Reviewer #2: Yes

********** 

6. Review Comments to the Author

Please use the space provided to explain your answers to the questions above. You may also include additional comments for the author, including concerns about dual publication, research ethics, or publication ethics. (Please upload your review as an attachment if it exceeds 20,000 characters)

Reviewer #2: Reviewer’s comments

MIDWIFE OR DOCTOR LEADER TO IMPLEMENT A NATIONAL GUIDELINE IN BABIES ON POSTNATAL WARDS (DesIGN): A CLUSTER- RANDOMISED, CONTROLLED, TRIAL.

General comments

This is an interesting paper trying to look at implementation of a clinical practice guideline (treating hypoglycemia in later preterm and term neonates with oral dextrose gel) using either a doctor or a midwife as implementation local leader. The authors had a clear aim as well as a well-defined hypothesis. The methodology is robust, and the results support the conclusion that doctors, and midwives are equally effective in implementing a clinical practice guideline.

The study was completed in 2017. Can the authors specify why there was a delay of nearly 5 years in publishing these data?

Specific comments

Abstract:

1. Authors have specified secondary outcomes as change in proportion from before to 6 months after implementation. But they have not discussed the results of that in the abstract. A simple sentence with that respect can complete the flow.

Methods

1. It would be helpful for the readers if the authors add their various components of Implementation tool kit (such as flow charts, pocket cards, posters etc) as Supplementary materials.

2. Statistical Analysis: The authors have mentioned that data are represented as Odd’s ratio. But the Tables show them as mean difference.

Results, Tables and Figures

1. Authors are advised to define the abbreviations at various places such as Table 2 (NICU, SD, IQR), Table 3 (SD, NICU), Line 288 - ICC

2. Lines 250-251: Authors have mentioned that the baseline characteristics were similar between the two groups, but they have not mentioned the related p values in Table 1.

3. Lines 261-262: 12/15 hospitals used dextrose gel. But Table 1 shows 10 hospitals used dextrose gel prior to implementation.

4. Authors can shorten non-significant p values as single decimal value (e.g. Line 29 – p value mentioned as 0.11. It can be mentioned as p= 0.1.). Also in Table 3.

Discussion

1. The flow of discussion is acceptable.

2. The authors have mentioned the strengths and limitations appropriately.

3. Authors can add some more discussions regarding barriers to implementation of guidelines and various other methods of implementation. (Rosa RG, et al. J Crit Care. 2020).

Conclusions:

1. Line 417: Can modify as “equally effective”

Grammatical Errors:

1. Lines 363- 364: Kindly re-frame as “there was no improvement in…”

********** 

7. PLOS authors have the option to publish the peer review history of their article (what does this mean?). If published, this will include your full peer review and any attached files.

If you choose “no”, your identity will remain anonymous but your review may still be made public.

**Do you want your identity to be public for this peer review?** For information about this choice, including consent withdrawal, please see our Privacy Policy.

Reviewer #2: No

**********

[NOTE: If reviewer comments were submitted as an attachment file, they will be attached to this email and accessible via the submission site. Please log into your account, locate the manuscript record, and check for the action link "View Attachments". If this link does not appear, there are no attachment files.]

While revising your submission, please upload your figure files to the Preflight Analysis and Conversion Engine (PACE) digital diagnostic tool, https://pacev2.apexcovantage.com/. PACE helps ensure that figures meet PLOS requirements. To use PACE, you must first register as a user. Registration is free. Then, login and navigate to the UPLOAD tab, where you will find detailed instructions on how to use the tool. If you encounter any issues or have any questions when using PACE, please email PLOS at figures@plos.org. Please note that Supporting Information files do not need this step.

# Author comment: Author response to Decision Letter 2

**DOI:** 10.1371/journal.pone.0291784.r006

30 Jul 2023

31 July 2023

Dear Editor,

 Thank you for the opportunity to respond do the reviewer, our comments are below,

1. The study was completed in 2017. Can the authors specify why there was a delay of nearly 5 years in publishing these data?

Data collection was finished at the end of 2017, data cleaning, writing of the Statistical Analysis Plan and data analysis were undertaken in 2018, and the paper written up for publication in 2019, with some delays then due to COVID at the beginning of 2020. The paper was first submitted for publication in June 2020, and has been submitted to two journals prior to submission to PLOS ONE. Editors seem to have had difficulty in finding reviewers, for example this manuscript was first sent to PLOS One in April 2022, the initial response to reviewers’ comments was submitted in June 2022 and the second response in Oct 2022, subsequently it has taken 8 months for the journal to respond, and it looks like they needed to find a replacement reviewer – thank you for reviewing the paper. 

Specific comments

Abstract:

1. Authors have specified secondary outcomes as change in proportion from before to 6 months after implementation. But they have not discussed the results of that in the abstract. A simple sentence with that respect can complete the flow.

We agree that this would improve the flow, but unfortunately it takes the abstract over the word limit of 300 words, so we have removed the mention of the secondary outcome in the abstract. 

Methods

1. It would be helpful for the readers if the authors add their various components of Implementation tool kit (such as flow charts, pocket cards, posters etc) as Supplementary materials.

The flow chart poster and pocket card have been added to the Supplementary materials. 

2. Statistical Analysis: The authors have mentioned that data are represented as Odd’s ratio. But the Tables show them as mean difference.

We have clarified the statistical analysis, page 11 line 236. The data are mean difference in Table 3 and Odds ratio and mean difference in the Supplementary Table. 

Results, Tables and Figures

1. Authors are advised to define the abbreviations at various places such as Table 2 (NICU, SD, IQR), Table 3 (SD, NICU), Line 288 – ICC

These abbreviations have been defined, including ICC in it’s first use on line 193.

2. Lines 250-251: Authors have mentioned that the baseline characteristics were similar between the two groups, but they have not mentioned the related p values in Table 1.

It is not a meaningful practice to present p values for the baseline characteristics of a randomised controlled trial. (Bland, J.M., Altman, D.G. Comparisons against baseline within randomised groups are often used and can be highly misleading. Trials 12, 264 (2011). https://doi.org/10.1186/1745-6215-12-264)

3. Lines 261-262: 12/15 hospitals used dextrose gel. But Table 1 shows 10 hospitals used dextrose gel prior to implementation.

As use of oral dextrose gel was a stratification variable, we collected this data point prior to randomisation by asking clinical leads if their hospital used oral dextrose gel, and were told that 10/15 hospitals used gel. However, during the pre-implementation data collection, which occurred after randomisation, this had increased to 12/15 hospitals having one or more babies that were treated with oral dextrose gel. This has been clarified in the Discussion, line 405-7. 

4. Authors can shorten non-significant p values as single decimal value (e.g. Line 29 – p value mentioned as 0.11. It can be mentioned as p= 0.1.). Also in Table 3.

We would prefer to keep the p values at two decimal places but would be happy to change this in the editing phase if this is the style of the journal.

Discussion

1. The flow of discussion is acceptable.

2. The authors have mentioned the strengths and limitations appropriately.

Thank you

3. Authors can add some more discussions regarding barriers to implementation of guidelines and various other methods of implementation. (Rosa RG, et al. J Crit Care. 2020).

Thank you for this reference. We have added a paragraph in the Discussion on barriers to implementation, lines 378-385. 

Conclusions:

1. Line 417: Can modify as “equally effective”

This has been changed as suggested. 

Grammatical Errors:

1. Lines 363- 364: Kindly re-frame as “there was no improvement in…”

This has been changed as suggested.

# Peer review: Decision Letter 3

**DOI:** 10.1371/journal.pone.0291784.r007

**Authors:** Sourabh Dutta

18 Sep 2023

Midwife or doctor leader to implement a national guideline in babies on postnatal wards (DesIGN): a cluster-randomised, controlled, trial.

PONE-D-22-10106R3

Dear Dr. Alsweiler,

We’re pleased to inform you that your manuscript has been judged scientifically suitable for publication and will be formally accepted for publication once it meets all outstanding technical requirements.

Within one week, you’ll receive an e-mail detailing the required amendments. When these have been addressed, you’ll receive a formal acceptance letter and your manuscript will be scheduled for publication.

An invoice for payment will follow shortly after the formal acceptance. To ensure an efficient process, please log into Editorial Manager at http://www.editorialmanager.com/pone/, click the 'Update My Information' link at the top of the page, and double check that your user information is up-to-date. If you have any billing related questions, please contact our Author Billing department directly at authorbilling@plos.org.

If your institution or institutions have a press office, please notify them about your upcoming paper to help maximize its impact. If they’ll be preparing press materials, please inform our press team as soon as possible -- no later than 48 hours after receiving the formal acceptance. Your manuscript will remain under strict press embargo until 2 pm Eastern Time on the date of publication. For more information, please contact onepress@plos.org.

Kind regards,

Sourabh Dutta

Academic Editor

PLOS ONE

Additional Editor Comments (optional):

Reviewers' comments:

Reviewer's Responses to Questions

**Comments to the Author**

1. If the authors have adequately addressed your comments raised in a previous round of review and you feel that this manuscript is now acceptable for publication, you may indicate that here to bypass the “Comments to the Author” section, enter your conflict of interest statement in the “Confidential to Editor” section, and submit your "Accept" recommendation.

Reviewer #1: All comments have been addressed

Reviewer #2: All comments have been addressed

**********

2. Is the manuscript technically sound, and do the data support the conclusions?

The manuscript must describe a technically sound piece of scientific research with data that supports the conclusions. Experiments must have been conducted rigorously, with appropriate controls, replication, and sample sizes. The conclusions must be drawn appropriately based on the data presented. 

Reviewer #1: (No Response)

Reviewer #2: Yes

**********

3. Has the statistical analysis been performed appropriately and rigorously? 

Reviewer #1: (No Response)

Reviewer #2: Yes

**********

4. Have the authors made all data underlying the findings in their manuscript fully available?

The PLOS Data policy requires authors to make all data underlying the findings described in their manuscript fully available without restriction, with rare exception (please refer to the Data Availability Statement in the manuscript PDF file). The data should be provided as part of the manuscript or its supporting information, or deposited to a public repository. For example, in addition to summary statistics, the data points behind means, medians and variance measures should be available. If there are restrictions on publicly sharing data—e.g. participant privacy or use of data from a third party—those must be specified.

Reviewer #1: (No Response)

Reviewer #2: Yes

**********

5. Is the manuscript presented in an intelligible fashion and written in standard English?

PLOS ONE does not copyedit accepted manuscripts, so the language in submitted articles must be clear, correct, and unambiguous. Any typographical or grammatical errors should be corrected at revision, so please note any specific errors here.

Reviewer #1: (No Response)

Reviewer #2: Yes

**********

6. Review Comments to the Author

Please use the space provided to explain your answers to the questions above. You may also include additional comments for the author, including concerns about dual publication, research ethics, or publication ethics. (Please upload your review as an attachment if it exceeds 20,000 characters)

Reviewer #1: (No Response)

Reviewer #2: The authors have answered all the questions appropriately and I feel the article in its current form can be accepted for publication.

**********

7. PLOS authors have the option to publish the peer review history of their article (what does this mean?). If published, this will include your full peer review and any attached files.

If you choose “no”, your identity will remain anonymous but your review may still be made public.

**Do you want your identity to be public for this peer review?** For information about this choice, including consent withdrawal, please see our Privacy Policy.

Reviewer #1: No

Reviewer #2: No

**********

# Editor report: Acceptance letter

**DOI:** 10.1371/journal.pone.0291784.r008

**Authors:** Sourabh Dutta

19 Sep 2023

PONE-D-22-10106R3 

Midwife or doctor leader to implement a national guideline in babies on postnatal wards (DesIGN): a cluster-randomised, controlled, trial. 

Dear Dr. Alsweiler:

I'm pleased to inform you that your manuscript has been deemed suitable for publication in PLOS ONE. Congratulations! Your manuscript is now with our production department. 

If your institution or institutions have a press office, please let them know about your upcoming paper now to help maximize its impact. If they'll be preparing press materials, please inform our press team within the next 48 hours. Your manuscript will remain under strict press embargo until 2 pm Eastern Time on the date of publication. For more information please contact onepress@plos.org.

If we can help with anything else, please email us at plosone@plos.org. 

Thank you for submitting your work to PLOS ONE and supporting open access. 

Kind regards, 

PLOS ONE Editorial Office Staff

on behalf of

Dr. Sourabh Dutta 

Academic Editor

PLOS ONE
